# Looking for the bird Kiss: evolutionary scenario in sauropsids

**DOI:** 10.1186/1471-2148-14-30

**Published:** 2014-02-19

**Authors:** Jérémy Pasquier, Anne-Gaëlle Lafont, Karine Rousseau, Bruno Quérat, Philippe Chemineau, Sylvie Dufour

**Affiliations:** 1 Muséum National d’Histoire Naturelle, UMR Biology of Aquatic Organisms and Ecosystems (BOREA), CNRS 7208, IRD 207, UPMC, Sorbonne Universités, F-75231 Paris Cedex 05, France; 2 Université Paris Diderot, Sorbonne Paris Cité, Unité Biologie Fonctionnelle et Adaptative (BFA), UMR8521 CNRS, U1133 Inserm, F-75013 Paris, France; 3 INRA, CNRS, Université Tours, Haras Nationaux, UMR 6175 Physiologie de la Reproduction et des Comportements (PRC), F-37380 Nouzilly, France

**Keywords:** Kisspeptin, Kiss receptor, Phylogeny, Synteny, Amniotes, Sauropsids, Birds

## Abstract

**Background:**

The neuropeptide Kiss and its receptor KissR are key-actors in the brain control of reproduction in mammals, where they are responsible for the stimulation of the activity of GnRH neurones. Investigation in other vertebrates revealed up to 3 *Kiss* and 4 *KissR* paralogs, originating from the two rounds of whole genome duplication in early vertebrates. In contrast, the absence of *Kiss* and *KissR* has been suggested in birds, as no homologs of these genes could be found in current genomic databases. This study aims at addressing the question of the existence, from an evolutionary perspective, of the Kisspeptin system in birds. It provides the first large-scale investigation of the Kisspeptin system in the sauropsid lineage, including ophidian, chelonian, crocodilian, and avian lineages.

**Results:**

Sauropsid Kiss and KissR genes were predicted from multiple genome and transcriptome databases by TBLASTN. Phylogenetic and syntenic analyses were performed to classify predicted sauropsid Kiss and KissR genes and to re-construct the evolutionary scenarios of both gene families across the sauropsid radiation.

Genome search, phylogenetic and synteny analyses, demonstrated the presence of two *Kiss* genes (*Kiss1* and *Kiss2* types) and of two *KissR* genes (*KissR1* and *KissR4* types) in the sauropsid lineage. These four genes, also present in the mammalian lineage, would have been inherited from their common amniote ancestor. In contrast, synteny analyses supported that the other *Kiss* and *KissR* paralogs are missing in sauropsids as in mammals, indicating their absence in the amniote lineage. Among sauropsids, in the avian lineage, we demonstrated the existence of a *Kiss2-like* gene in three bird genomes. The divergence of these avian *Kiss2-like* sequences from those of other vertebrates, as well as their absence in the genomes of some other birds, revealed the processes of *Kiss2* gene degeneration and loss in the avian lineage.

**Conclusion:**

These findings contribute to trace back the evolutionary history of the Kisspeptin system in amniotes and sauropsids, and provide the first molecular evidence of the existence and fate of a *Kiss* gene in birds.

## Background

In 1996, kisspeptin, the product of the *Kiss1* gene, was first discovered as an anti-metastatic peptide in human carcinoma [1]. The *Kiss1* gene encodes a kisspeptin-precursor secondarily processed to give size-variants of kisspeptins, including kisspeptin-54 [Kp(54)], kisspeptin-14 [Kp(14)] and kisspeptin-13 [Kp(13)], in human [2,3]. All these kisspeptin size-variants encompass the C-terminal 10-amino acid sequence [Kp(10)], which represents the minimal sequence for bioactivity. This Kp(10) sequence also presents the pecularity to be highly conserved among vertebrates, while the other part of the precursor sequence is highly variable. Kisspeptins belong to the RF-amide peptide family which also includes the neuropeptide FF (NPFF), the gonadotropin-inhibiting hormone (GnIH), the prolactin-releasing peptide (PrRP) and the 26RFa peptides [4]. In 1999, *GPR54*, a gene encoding an orphan G-protein coupled receptor, was cloned in the rat [5]. It was not until 2001 that GPR54 was identified as the cognate receptor of the kisspeptins [2,3].

Both kisspeptin (Kiss) and its receptor (KissR) were demonstrated as crucial players of the reproductive function in mammals [6-11]. They act upstream in the gonadotropic axis by activating gonadotropin-releasing hormone (GnRH) neurons and are considered as major puberty gatekeepers and reproduction regulators [12]. Mutations or targeted deletions of *Kiss* or *KissR* resulted in hypogonadotropic hypogonadism in human and rodents [6-10]. This pathology is characterised by the failure of the reproductive function due to low circulating levels of gonadotropin hormones (LH and FSH), inducing low plasmatic levels of sex steroids including estradiol (E2), testosterone and progesterone (for review: [13]). In contrast, overexpression of kisspeptin can induce precocious puberty onset in human and rodents [14-16].

Since their discovery in mammals, the kisspeptin systems (Kiss and its receptor KissR) have been identified in most vertebrate groups including cyclostomes, chondrichtyans, teleosts, amphibians and sauropsids [17-19]. Phylogenetic and syntenic analyses [17,18,20,21] revealed that three paralogous genes encode the current vertebrate kisspeptins, *i.e. Kiss1*, *Kiss2* and *Kiss3* genes, and up to four paralogous genes encode their receptors, *i.e. KissR1*, *KissR2*, *KissR3* and *KissR4* genes, according to the recent classifications by Pasquier *et al *[17,21]. This *Kiss* and *KissR* diversity likely arose from the two successive rounds of whole genome duplication (1R and 2R) [17,21] that have occurred in early vertebrates [22,23]. Following these events, the evolutionary history of *Kiss* and *KissR* was marked by multiple gene loss events in the various vertebrate lineages [17,21]. Notably, due to massive gene loss, there is no impact of the teleost specific third round of genome duplication (3R) on the current number of Kiss or KissR in teleosts [17,21]. Strikingly among sauropsids, a complete loss of the kisspeptin system may have occurred in birds, as suggested by the lack of *Kiss* and *KissR* homologs in the current bird genomic databases [4,17-21,24].

Nevertheless, some immunocytochemical and experimental studies suggest the existence of a kisspeptin system in birds. Previous studies, using polyclonal antibodies against human Kp(10), have reported the observation of Kiss immunoreactivity in mallard duck hypothalamus [25] and hen cultured granulosa cells [26]. Even if these results have to be considered with caution since they could reflect immune cross-reaction with other RF-amide peptides, effects of human Kp(10) on reproductive function [25-27], lipid metabolism [28] and food intake [29] have also been reported in birds. Concerning the reproductive function, in adult mallard drake, central administration of human Kp(10) was able to increase the plasma concentration of LH [25]. In juvenile female quail, daily peripheral injections of human Kp(10) induced an anticipated onset of egg-laying, an accelerated growth of the reproductive organs and an increase in E2 secretion [27]. The same treatments also increased *cGnRH-I*, *GnRH-Receptor II* and *LHβ* mRNA expressions, while they decreased *GnIH* and *FSHβ* expressions [27]. *In vitro*, human Kp(10) treatment increased progesterone secretion in cultured chicken ovarian granulosa cells from preovulatory follicles [26]. In contrast, in adult male chicken, peripheral injections of human Kp(10) led to decreased circulating testosterone level [30]. The effects of exogenous Kp(10) treatments on the bird gonadotropic axis could be obtained using other RF-amide peptides such as human 26RFa suggesting that they could be non-specific [30]. Furthermore, it has recently been suggested that this inhibitory effect of exogenous Kp(10) on testosterone levels could be mediated by GnIH receptor in bird, since kisspeptin was shown to bind to this receptor [31].

As functional approaches suggest that exogenous kisspeptins could exert physiological effects on birds while no *Kiss* or *KissR* has been evidenced in the different bird genomic databases so far, the question of the existence of a kisspeptin system in birds remains open. In order to address this question in an evolutionary context, we choose to investigate the *Kiss* and *KissR* gene diversity in the genomic databases from representatives of different sauropsid groups, including ophidian, chelonian, crocodilian, and bird genomes. This integrated approach enabled us to improve the evolutionary history of both *Kiss* and *KissR* families in the sauropsid lineage and to demonstrate, for the first time, the presence of a *Kiss2-like* gene in the genome of three different bird species.

## Results and discussion

### Prediction of sauropsid *Kiss*

To further assess the *Kiss* diversity, we investigated the presence of these genes in the genomes from various sauropsid groups: the Indian python as representative of ophidians; the painted turtle and the Chinese (soft-shell) turtle as representatives of the chelonians; the American alligator, the saltwater crocodile and the Indian garial as representatives of crocodilians; the chicken, the turkey, the mallard duck, the collared flycatcher, the zebra finch, the medium ground finch, the rock pigeon, the saker falcon, the peregrine falcon, the Tibetan ground-tit, the budgerigar and the Puerto-Rican parrot as representative of birds.

Considering that the *Kiss* gene sequences are highly variable among species except for the sequence encoding the Kp(10) and its flanking proteolytic cleavage and alpha-amidation site, we focused our predictions on the open reading frame (ORF) containing these sequences. Using various vertebrate sequences as query, we performed TBLASTN in the above-mentioned eighteen sauropsid genomes (including twelve bird genomes), resulting in the identification of ORFs containing conserved sequences encoding Kp(10).

#### *Two Kiss genes in ophidians*

**Figure 1:**
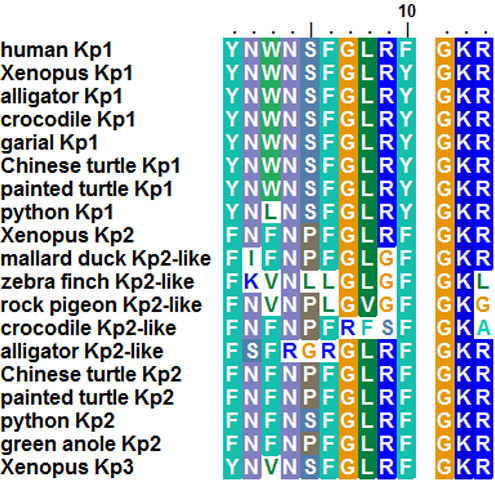
**Amino-acid sequences of Kisspeptin 10 (Kp(10)) and their proteolytic and alpha-amidation sites from various vertebrates, including novel predicted sequences from sauropsids.** Amino acids are coloured according to their physico-chemical properties. The C-terminal GKR motif represents the typical proteolytic and alpha-amidation site. Striking substitutions in bird and crocodilian sequences are highlighted by white background.

Two ORFs containing the sequences encoding Kp(10) were found in the Indian python genome. These two ORFs are 369 bp and 390 bp long, respectively (Additional file 1: Figure S1A). Once translated, each of them leads to a peptidic sequence encompassing a putative Kp(10): YNLNSFGLRY [Indian python Kp1(10)] and FNFNSFGLRF [Indian python Kp2(10)], respectively (Figure 1). The C-terminal ends of these two sequences are followed by a GKR motif (Figure 1). The sequences “X-G-Basic-Basic” or “X-G-Basic-Stop” are characteristic of the conserved proteolytic cleavage and alpha-amidation sites of neuropeptides [32]. *In silico* characterization of putative N-terminal proteolytic cleavage sites in the two translated ORFs led to the prediction of putative mature peptides of 46 a.a. long [Indian python Kp1(46)], 53 a.a. long [Indian python Kp2(53)] and 12 a.a. long [Indian python Kp2(12)] (Additional file 1: Figure S1 and Additional file 2: Figure S2).

#### *Two Kiss genes in chelonians*

Two ORFs containing the sequences encoding Kp(10) were found in each turtle genome. These ORFs are 528 bp and 465 bp long in both species (Additional file 1: Figure S1B-C). Each of them encoded a putative Kp(10) followed by a typical C-terminal GKR motif: YNWNSFGLRY [painted turtle Kp1(10) and Chinese turtle Kp1(10)] and FNFNPFGLRF [painted turtle Kp2(10) and Chinese turtle Kp2(10)] (Figure 1). *In silico* characterization of N-terminal proteolytic cleavage sites in the four ORFs led to the prediction of putative mature peptides of 48 a.a. long [painted turtle Kp1(48) and Chinese turtle Kp1(48)] and 53 a.a. long [painted turtle Kp2(53) and Chinese turtle Kp2(53)] (Additional file 1: Figure S1 and Additional file 2: Figure S2).

#### *One Kiss gene in crocodilians*

One ORF containing the sequence encoding Kp(10) was found in each of the three crocodilian genomes. Those ORFs are 567 bp, 402 bp and 348 bp long for alligator, crocodile and garial, respectively (Additional file 1: Figure S1D-F). All of them encoded a sequence encompassing a putative Kp(10): YNWNSFGLRY [alligator Kp1(10), crocodile Kp1(10) and garial Kp1(10)] (Figure 1). The three peptidic sequences are followed by a typical GKR motif (Figure 1)*. In silico* characterization of N-terminal proteolytic cleavage sites in the three ORFs led to the prediction of putative mature peptides of 45 a.a. long [alligator Kp1(45) and crocodile Kp1(45)], 43 a.a. long [garial Kp1(45)] and 29 a.a. long [alligator Kp1(29), crocodile Kp1(29), and garial Kp1(29)] (Additional file 1: Figure S1 and Additional file 2: Figure S2).

In addition to our findings, Osugi *et al*. recently identified a *Kiss-like* sequence in crocodile and alligator genomes, with striking amino-acid substitutions in positions 7, 8 and 9 for the crocodile Kp(10)-like and positions 2, 4, 5 and 6 for the alligator Kp(10)-like (Figure 1). The authors suggest that the crocodilian *Kiss-like* gene could be a pseudogene belonging to the Kiss2 type that may be non-functional because of the presence of premature stop-codons upstream of the coding sequence of Kp2(10)-like [33].

#### *One Kiss-like gene in birds*

**Figure 2:**
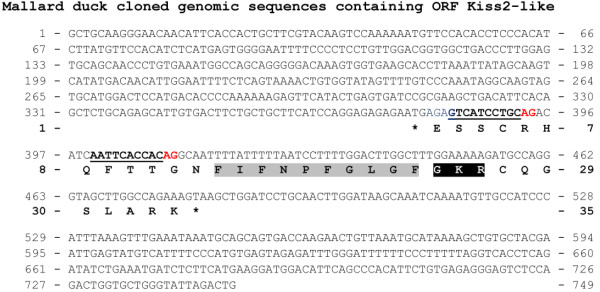
**Mallard duck cloned genomic sequence encompassing Kiss2-like ORF.** Nucleotides (top) are numbered from 5′ to 3′. The amino-acid residues (bottom) are numbered beginning with the first residue in the open reading frame (ORF). The asterisks (*) indicate the stop codons delineating the ORF. The predicted Kp2(10) peptide is shaded in grey and the predicted C-terminal proteolytic and alpha-amidation site is shaded in black. The predicted putative splice acceptor sites (AG) located between the stop codon and the region encoding the Kp(10) are coloured in red, and the preceding T/C rich sequences are underlined.

At first, the TBLASTN performed in bird genomic databases, using previously characterized kisspeptin sequences and newly described sauropsid mature kisspeptins as queries, resulted in no hit except for the mallard duck genome, pointing out a 99 bp long ORF. We then amplified and cloned the genomic region encompassing the corresponding mallard duck ORF (Figure 2). It encodes a peptidic sequence presenting the following 10 a.a.-sequence: FIFNPFGLGF (Figures 1 and 2). The C-terminal end of this sequence is followed by a typical GKR motif (Figures 1 and 2). The mallard duck Kp(10)-like sequence shares 40% identity with crocodilian, chelonian and ophidian Kp1(10) while it shares 80% identity with the anole Kp2(10), the painted turtle Kp2(10) and the Chinese turtle Kp2(10) (Figure 1). This is the first evidence of a Kiss-like sequence in birds.

The observed mismatches, between mallard duck Kp(10) and the other sauropsids Kp2(10), concern the highly conserved amino acids at the second and ninth positions of the 10 a.a.-sequence (Figure 1). Vertebrate Kp(10) usually present an asparagine (N) in second position whereas it is substituted by an isoleucine (I) in the mallard duck Kp(10). Similarly vertebrate Kp(10) also possess an arginine (R) in ninth position whereas it is substituted by a glycine (G) in the mallard duck Kp(10), turning the kisspeptin typical C-terminal RF (or RY) motif into a GF one. These two positions may not be critical for Kp(10) bioactivity. Indeed, alanine (A) substitution performed at one or the other of these two positions on human and rat Kp(10) induced no major change in bioactivity, although it decreased efficiency and binding property [34-36]. Nevertheless, to our knowledge, no Kp(10) with both substitutions has been tested. Thus, it could be of particular interest, for a future study, to test, *in vitro* and *in vivo*, the mallard duck Kp(10).

The sequence encoding the mallard duck Kp(10)-like is encompassed in a small ORF (99 bp), and located 36 nucleotides downstream of a stop codon (Figure 2). In contrast to the other longer ORFs identified in this study, no classical di-basic N-terminal end proteolytic cleavage site could be predicted in the translated mallard duck *Kiss-like* ORF. This did not allow the prediction of a putative mature peptide. In addition to the amino-acid substitutions in the Kp(10)-like sequence, these observations suggest that the mallard duck *Kiss-like* gene is degenerated and could be a non-functional pseudogene, as suggested by Osugi *et al.* for crocodilian *Kiss2-like* pseudogene [33].

Nevertheless, the presence of two putative splice acceptor sites, between the 5′ stop codon and the sequence encoding the mallard duck Kp(10)-like (Figure 2), opens the possibility that the mallard duck *Kiss-like* gene possesses a different exon/intron structure compared to the other vertebrate *Kiss* genes. However no potential exon coding for a signal peptide has been predicted so far that could support this hypothesis. Similarly, it is notable that the coelacanth Kp3(10) is encoded by a 81 bp-long ORF, that also suggests a different structure or a loss of function for this gene [17].

As a second step, using the mallard duck Kiss-like sequence as query, we performed TBLASTN in the other bird genomic databases. The results of this second TBLASTN series pointed out a 279 bp-long ORF in the zebra finch genome (Additional file 1: Figure S1) and a 117 bp-long ORF in rock pigeon (Additional file 1: Figure S1). Each of them encodes a peptidic sequence encompassing the following 10 a.a.: FKVNLLGLGF and FNVNPLGVGF, respectively (Figure 1). These sequences share 70% identity with each other, 60% identity with mallard duck Kp(10), 50% and 60% identity, respectively, with the anole Kp2(10), and only 40% identity with the crocodilian and chelonian Kp1(10) (Figure 1). Both zebra finch and rock pigeon Kp(10) present a leucine (L) at their sixth positions, whereas other vertebrate Kp(10), present a phenylalanine (F) at the same position. It can be noted that alligator Kp2(10)-like presents a F to R substitution at this position. The substitution of this F has been described as critical for the rat Kp(10) tridimensional structure and bio-activity [36]. The Kp(10) sequences are followed by a GKL motif in C-terminal for the zebra finch and a GKG motif for the rock pigeon (Figure 1 and Additional file 1: Figure S1). They represent unusual proteolytic cleavage and alpha amidation site motifs among kisspeptins, which commonly are of the G-basic-basic type (i.e. GKR or GRR) or G-basic-stop type. As in mallard duck, the sequences encoding the zebra finch and the rock pigeon Kp(10)-like are located 18 and 33 nucleotides downstream a 5′ stop codon (Additional file 1: Figure S1). In contrast to the other longer ORFs identified in this study, but similarly to the mallard duck, no classical di-basic N-terminal end proteolytic cleavage site were present in the translated zebra finch and the rock pigeon *Kiss-like* ORFs, preventing the prediction of any putative mature peptide.

The presence of three putative splice acceptor sites, between the 5′ stop codon and the sequence encoding the rock pigeon Kp(10)-like (Additional file 1: Figure S1), allows the possibility that the rock pigeon *Kiss-like* gene possesses a different exon/intron structure, as proposed for mallard duck *Kiss-like* gene. In contrast, no putative splice acceptor site has been predicted between the 5′ stop codon and the sequence encoding the zebra finch Kp(10)-like (Additional file 1: Figure S1). In addition, no potential exon coding for a signal peptide could be predicted in this study, neither for rock pigeon *Kiss-like* nor zebrafinch *Kiss-like*. As suggested above for the mallard duck sequence, this could reflect either a different structure or a loss of function for these genes.

Finally, using the zebra finch and rock pigeon Kiss-like sequences as queries, we performed a novel TBLASTN search in the other genomic databases of birds, including chicken. This third TBLASTN series returned no hit, suggesting that the *Kiss-like* genes of the other birds are even more degenerated than those found in the mallard duck, zebra finch and rock pigeon genomes.

### Classification of the sauropsid Kiss

We recently demonstrated, by synteny analysis, that gnathostome *Kiss* can be classified into three different groups, *Kiss1*, *Kiss2* and *Kiss3*, respectively [17] which likely result from the 1R and 2R events in early vertebrates. The putative fourth *Kiss* (*Kiss4*) has not been discovered in any current vertebrate species suggesting an early loss after the 2R [17]. In sauropsids, our previous study demonstrated that green anole possesses only a *Kiss2* gene [17] and the recent study by Osugi *et al.* demonstrated that chelonians possess a *Kiss2* gene and crocodilian a *Kiss2-like* pseudogene [33]. In the present study, we performed phylogenetic and syntenic analyses in order to further identify and classify the newly predicted sauropsid *Kiss* genes.

#### *Phylogenetical analysis of Kiss genes*

**Figure 3:**
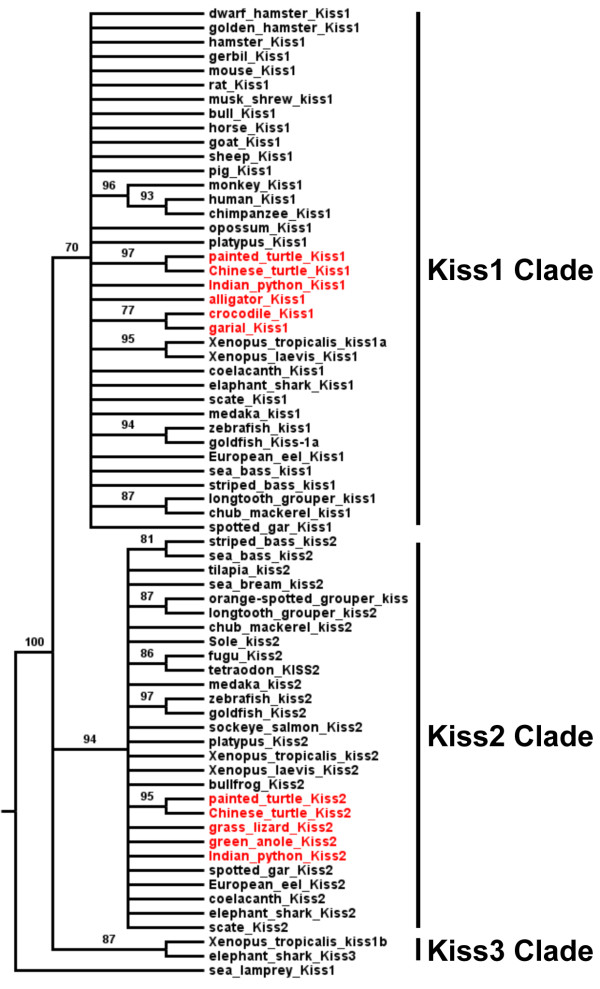
**Consensus phylogenetic tree of vertebrate kisspeptins.** This phylogenetic tree was constructed based on the amino-acid sequences of putative mature kisspeptins (for the alignment and references of sequences see Additional files 2 and 3: Figure S2 and Table S1) using the Neighbour Joining method with 1,000 bootstrap replicates. The number shown at each branch node indicates the bootstrap value (%); only values and branching above 70% are indicated. The tree was rooted using the sea lamprey Kiss1 sequence. The sauropsid kisspeptins are in red.

Based on an alignment of 68 predicted long mature kisspeptin (Additional files 2 and 3: Figure S2 and Table S1), and assuming sea lamprey Kiss1 sequence as the out-group, a phylogenetic tree was generated (the list of the sequences and accession numbers is provided in Additional file 2: Figure S2). Our *in silico* prediction of N-terminal proteolytic cleavage sites did not enable us to predict mature kisspeptin from the bird *Kiss-like* (this study), the crocodilian *Kiss2-like *[33] and the coelacanth *Kiss3*[17], which were excluded from this analysis. As shown in Figure 3, gnathostome mature kisspeptins cluster into three clades, Kiss1, Kiss2 and Kiss3, which are supported by significant bootstrap values: 70%, 94% and 87%, respectively.

The Kiss1 clade encompasses the chondrichtyan and actinopterygian Kiss1 sequences including the spotted gar and teleost Kiss1 sequences. The Kiss1 clade also encompasses the sarcopterygian Kiss1 sequences including the coelacanth, the amphibian and various mammalian Kiss1 sequences and some predicted sauropsid Kiss sequences among which the python Kiss1, the painted turtle Kiss1, the Chinese turtle Kiss1, the alligator Kiss1, the crocodile Kiss1 and the garial Kiss1 sequences (Figure 3). These results provide the first evidence for the presence of orthologs to mammal *Kiss1* in sauropsids.

The Kiss2 clade clusters the chondrichtyan Kiss2 sequences, the actinopterygian Kiss2 sequences, including the spotted gar Kiss2 and various teleost Kiss2 sequences. It also encompasses the sarcopterygian Kiss2 sequences including the coelacanth Kiss2, the Amphibian Kiss2, the platypus Kiss2 and predicted sauropsid Kiss sequences among which the python Kiss2, the painted turtle Kiss2 and the Chinese turtle Kiss2 in addition to the already described grass lizard Kiss2 and green anole Kiss2 sequences (Figure 3).

The Kiss3 clade clusters one amphibian sequence (*Xenopus tropicalis* Kiss1b) with one chondrichtyan (elephant shark Kiss3) (Figure 3). Thus, this phylogenetic analysis strengthens the existence of three kisspeptin clades among gnathostomes. It also demonstrates that the predicted Kiss, from the sauropsid genomes investigated so far, belong to the Kiss1 or Kiss2 clades.

#### *Syntenic analysis of Kiss genes*

**Figure 4:**
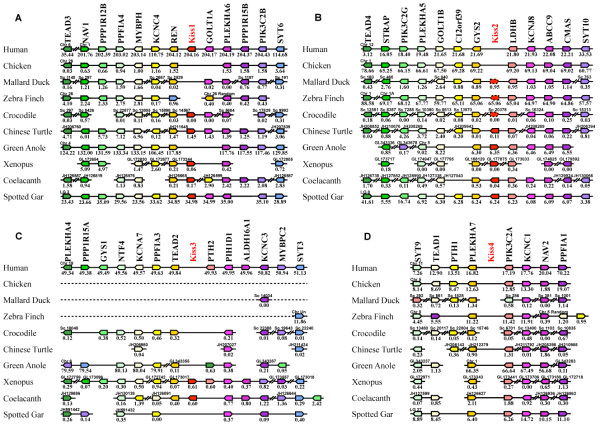
**Conserved genomic synteny of osteichtyan *Kiss *genes.** Genomic synteny maps comparing the orthologs of *Kiss1***(A)**, *Kiss2***(B)**, *Kiss3***(C)**, *Kiss4***(D)** and their neighbouring genes. *Kiss* genes are named according to our proposed nomenclature (*Kiss1* to *Kiss4*, [17]). The other genes are named after their human orthologs according to the Human Genome Naming Consortium (HGNC). Orthologs of each gene are shown in the same color. The direction of arrows indicates the gene orientation, with the ID of the genomic segment indicated above and the position of the gene (in 10^-6^ base pairs) indicated below. The full gene names and detailed genomic locations are given in Additional file 7: Table S3.

In order to test the results obtained with the phylogenetic analysis, and to further understand the evolutionary history of *Kiss* genes in the sauropsid lineage, we performed a syntenic analysis of the *Kiss* neighbouring genes in representative sauropsid genomes. As we had already demonstrated the orthology relationships of the *Kiss* genes for some sarcopterygian and actinopterygian species by synteny analyses [17], we used the same species as references in the current study. Thus, for the present analysis (Figure 4), we focused on one actinopterygian (spotted gar) and the following sarcopterygian representatives: basal sarcopterygian (coelacanth), amphibian (Xenopus), squamate (anole), chelonian (Chinese turtle), crocodilian (crocodile), bird (chicken, mallard duck and zebra finch) and mammals (human).

The human *Kiss1*, crocodile *Kiss1*, Chinese turtle *Kiss1*, *Xenopus Kiss1a,* coelacanth *Kiss1* and spotted gar *Kiss1* are positioned in genomic regions containing common loci, including *TEAD3*, *NAV1, PPP1R12B, PPFIA4, MYBPH, KCNC4, REN, GOLT1A*, *PLEKHA6, PPP1R15B, PIK3C2B* and *SYT6* (Figure 4A). The painted turtle *Kiss1*, alligator *Kiss1* and garial *Kiss1* are also located in the vicinity of those genes (data not shown). This supports the orthology relationship between all these *Kiss* genes, all considered as *Kiss1* genes. Syntenic analysis supports the absence of *Kiss1* gene in the chicken, mallard drake, zebra finch and green anole, although the above-mentioned neighbouring genes are present in their respective genomic databases (Figure 4A).

The *Kiss-like* genes of mallard duck and zebra finch, the Chinese turtle, green anole, coelacanth and spotted gar *Kiss2* genes are positioned in genomic regions containing common loci including *TEAD4, STRAP, PLEKHA5, GOLT1B*, *C12orf39, GYS2, LDHB, KCNJ8, ABCC9, CMAS* and *SYT10* (Figure 4B). The painted turtle *Kiss2* and the *Kiss-like* sequence of the rock pigeon are also located in the vicinity of these genes (data not shown). The crocodile *Kiss2-like* is located in an isolated scaffold, in the vicinity of the *LDHB* gene. This supports the orthology relationship between all these *Kiss*, considered as *Kiss2* genes, or as *Kiss2*-like genes in the case of crocodile, mallard drake, zebra finch and rock pigeon. The syntenic analysis suggests that chicken would not possess any *Kiss2* gene, although the above-mentioned neighbouring genes are present in their respective genomic databases (Figure 4B).

As already demonstrated, the coelacanth *Kiss3* and the *Xenopus Kiss1b* genes are positioned in genomic regions containing common loci, including *NTF4, KCNA7, PPFIA3, TEAD2, PIH1D1*, *ALDH16A1, KCNC3, MYBPC2* and *SYT3* (Figure 4C and [17]). This supports the orthology relationship between these two *Kiss* genes, both considered here as *Kiss3* genes. Syntenic analysis supports the absence of *Kiss3* gene in human, crocodile, and green anole, although the above-mentioned neighbouring genes are present in their respective genomic databases (Figure 4C). Syntenic analysis also indicates that the considered region is largely missing from the bird and turtle genomic databases likely due to incomplete genome sequencing (see below). Indeed, among the fourteen considered genes in the *Kiss3* syntenic region, only *KCNC3* gene is present in mallard drake genomic databases, only *SYT3* gene is present in zebra finch genomic databases and only *KCNA7, PIH1D1* and *SYT3* genes are present in Chinese turtle genomic databases (Figure 4C).

Our previous syntenic analysis allowed us to consider a fourth genomic region in the osteichthyan genomes that could encompass a *Kiss4* gene [17]. This was based on the observation that the three conserved genomic regions, presenting *Kiss* genes, also comprise paralogs from other gene families including *TEAD1/2/3/4*, *NAV1/2/3*, *PPFIA1/2/3/4*, *KCNC1/2/3/4*, *GOLT1A/B*, *PLEKHA4/5/6/7*, *PPP1R15A/B*, *PIK3C2A/B/G*, *SYT3/6/9/10*, *GYS1/2* and *PTH1/2* (Figure 4 and [17]). The members of these families are present among the three *Kiss* syntenic regions and also delineate a fourth conserved region (Figure 4D). However, *Kiss4* genes are completely missing from all osteichthyan genomes, including sauropsid genomes, investigated so far. This further supports the early loss of *Kiss4* gene after the 2R in vertebrates.

Until the present study, the *Kiss* gene diversity had been investigated in only a few sauropsid species and only *Kiss2* genes have been described [17,33]. *Kiss2* gene had been shown in squamate and chelonian representatives, and *Kiss2-like* gene had been found in crocodilian representatives while no *Kiss* gene could be found in birds [17,18,24,33]. Our results, based on multiple sauropsid genomes, represent the first large-scale investigation and classification of *Kiss* genes in sauropsids. We report here the existence of the *Kiss1* gene for the first time in the sauropsid lineage. We also provide the first demonstration of the existence of a *Kiss-like* gene in three bird genomes (mallard duck, zebra finch and pigeon). Based on synteny analyses, bird *Kiss-like* gene could be assigned to *Kiss2* type. This is in agreement with the higher sequence identity between bird Kp(10)-like and the other sauropsid Kp2(10). Synteny analyses also highlighted the absence of *Kiss1* and *Kiss4* genes in birds, while the corresponding genomic environment is well conserved. In contrast, our syntenic analysis revealed an urgent need for genomic information in the putative region of bird *Kiss3* (Figure 4C). In the human genome, this region is located in the chromosome 19q (HSA19q) (Figure 4C). It has been well documented that most parts of the homologous region of HSA19q are not represented in the whole genome shotgun reads and BAC libraries used to build the bird genome databases [37,38]. However, it has been demonstrated that this region does exist and is split into micro-chromosomes in the bird genomes [38,39]. These observations still leave open the possibility of the presence of a *Kiss3* gene in birds. To further investigate the *Kiss* existence in birds, we looked for evidence of *Kiss* mRNA in the released bird transcriptomic databases.

### Investigation of the *Kiss* existence in bird transcriptome databases

As kisspeptins are considered as neuropeptides, the *Kiss* gene expressions were mainly investigated in the brain of vertebrates (for reviews: [40,41]). In human, in addition to its cerebral expression, *Kiss1* transcript is found in placenta, intestine, testis, pancreas, spleen, kidney and liver [3,42]. In *Xenopus*, *Kiss1a* (*Kiss1*-type), *Kiss1b* (*Kiss3*-type) and *Kiss2* genes are expressed in brain, testis, heart and liver [18].

In order to investigate the potential existence of *Kiss* transcripts in birds, we performed TBLASTN searches in twelve released transcriptomic databases, including brain transcriptomes from ten different bird species. We also investigated chicken transcriptomes including embryo, muscle, brain and liver, and zebra finch transcriptomes including blood and spleen. Using as query the same sequences as already used for the search against the sauropsid genomic databases as well as the bird *Kiss2-like* sequences, we performed TBLASTN in the twelve bird transcriptomic databases, but they resulted in no hit. In contrast, TBLASTN search using other RF-amide sequences as query, allowed us to retrieve transcripts of the corresponding peptides including GnIH, NPFF, 26RFa and PrRP (data not shown). The absence of result concerning kisspeptin, in all released databases from a large number of species, supports the potential loss of functional *Kiss* gene in birds. To further understand whether *Kiss* genes have been lost in birds and to improve the *Kiss* evolutionary scenario, we focused on the diversity of newly described *Kiss* genes among the sauropsids.

### Evolutionary scenario of *Kiss* genes in the sauropsid lineage

**Figure 5:**
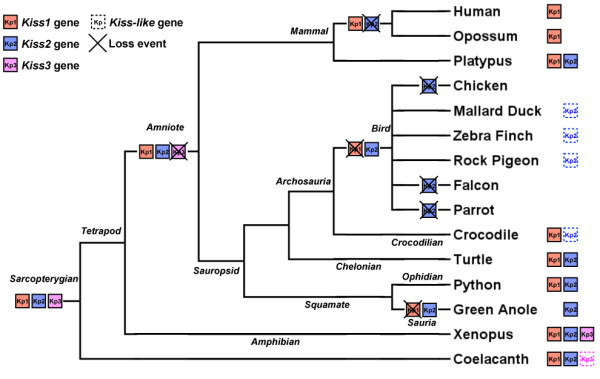
**Current status and proposed evolutionary history of *Kiss *genes among sarcopterygians.** The names of the current representative species of each vertebrate group are given at the end of the final branches, together with the symbol of the *Kiss* genes they possess. This evolutionary scenario assumes the most parsimonious hypothesis of the loss of *Kiss3* gene in the amniote common ancestor.

Our analyses demonstrate that *Kiss1* and/or *Kiss2* genes are present in the sauropsid lineage. No trace of *Kiss4* was found in agreement with the hypothesis that this loss-event would have taken place at the early stage of vertebrate evolution [17]. Among sarcopterygians, the *Kiss3* existence has been demonstrated in the coelacanth and the Xenopus genomes, [17,18] indicating that *Kiss3* gene has been inherited by the sarcopterygian ancestor and more recently by the tetrapod ancestor. No trace of *Kiss3* was found in the mammalian and sauropsid genomic databases, suggesting the loss of this gene in the amniote ancestor. However, the fact that the whole *Kiss3* genomic region is missing in the current bird genome databases, does not allow us to confirm the absence of *Kiss3* in birds by synteny analysis. Considering the alternative hypothesis of the presence of *Kiss3* in birds, the phylogenetic position of birds among amniotes implies that *Kiss3* gene would have been conserved in birds and lost independently in mammals, squamates (lizards and snakes), chelonians (turtles) and crocodilians lineages. This hypothesis represents four evolutionary events, as compared to only one event if considering the loss of *Kiss3* gene in the amniote ancestor (Figure 5). This scenario is therefore the most parsimonious and is also supported by the absence of *Kiss3* mRNA in the bird transcriptomic databases.

Following the most parsimonious scenario (Figure 5), only *Kiss1* and *Kiss2* genes are present in the sauropsid lineage. Both *Kiss1* and *Kiss2* are present in chelonians. *Kiss1* and *Kiss2* genes were also inherited by squamate ancestor since python still possesses both *Kiss1* and *Kiss2* genes, while *Kiss1* would have been lost in lizards. *Kiss1* and *Kiss2* genes may have been inherited by the crocodilian ancestor since garial, crocodile and alligator still possess a *Kiss1* gene and these two latter a *Kiss2-like* gene. In contrast, *Kiss1* gene would have been lost in birds, while the presence of a *Kiss2-like* gene in mallard duck, zebra finch and rock pigeon genomes suggests that *Kiss2* gene has been inherited by the bird ancestor (Figure 5).

The present investigation reveals the presence of a *Kiss-like* gene (*Kiss2*) for the first time in several bird genomes and suggests that it would be the only *Kiss* type remnant in this lineage. As discussed above, the characterized *Kiss2-like* genes are probably not functional. Furthermore, the sequence seems to be lacking in the other investigated bird genomes, likely reflecting a more advanced degenerating process.

These data suggest a loss of functional *Kiss* system in birds. However, as mentioned in the introduction, previous studies have reported the observation of immunoreactive Kiss cells in birds [25,26] and the effects of human kisspeptin on food intake [29], lipid metabolism [28] and reproductive function [25-27] in birds. Some of the effects of exogenous kisspeptin could be considered as non-specific as they can be obtained using other RF-amide peptides such as human 26RFa peptides [30,43] or the orexigenic neuropeptide Y (NPY) [44,45]. This suggests that exogenous kisspeptins are able to interact with, at least, one RF-amide receptor in birds. To date, no *KissR* gene or transcript has been identified in birds. That leads to the following question: do the birds still possess a *KissR* gene? In order to investigate this question, as we did for the *Kiss* genes, we focused our attention on the diversity and the evolutionary scenario of the *KissR* genes in the sauropsid lineage.

### Prediction of sauropsid *KissR* genes

Our previous studies demonstrated the existence of four *KissR* paralogs in vertebrates [21]. At that time, only green anole, chicken, turkey and zebra finch genomes had been investigated among sauropsids. Only one *KissR* had been found in green anole and none in those birds. To further assess the *KissR* diversity in sauropsids, we performed TBLASTN in the eighteen sauropsid genomes mentioned in section Prediction of sauropsid *KissR* genes using human KissR1, anole KissR2, Xenopus GPR54-1b (KissR3 according to our classification [21]) and predicted coelacanth KissR4 peptidic sequences as query. The TBLASTN analyses resulted in the identification of multiple new *KissR* genes in six out of eighteen investigated sauropsid genomes.

#### *Two KissR genes in ophidians*

Two *KissR* genes were predicted from the Indian python, each made of 5 exons and 4 introns (Additional file 4: Figure S3A-B). The putative transcripts encode two predicted proteins, *i.e.* python KissR1 and python KissR4 (named according to the phylogeny and synteny analyses, sections Phylogenetic analysis and Syntenic analysis of KissR genes), of 368-aa and 398-aa, respectively (Additional file 4: Figure S3).

#### *Two KissR genes in chelonians*

Two *KissR* genes were predicted from the painted turtle genome, each made of 5 exons and 4 introns (Additional file 4: Figure S3C-D). From the Chinese turtle genome one *KissR* gene was predicted, also made of 5 exons and 4 introns, while only 4 exons and 3 introns could be predicted for the second *KissR* gene, as the exon-1 was missing probably due to assembling problems (Additional file 4: Figure S3E-F). Transcripts encode predicted proteins, *i.e.* painted turtle KissR1 (359-aa), painted turtle KissR4 (388-aa), partial Chinese turtle KissR1 (282-aa) and Chinese turtle KissR4 (388-aa) (Additional file 4: Figure S3).

#### *One KissR gene in crocodilians*

One *KissR* gene, made of 5 exons and 4 introns, was predicted in crocodile and garial genomes encoding crocodile KissR1 (371-aa) and garial KissR1 (372-aa) (Additional file 4: Figure S3G-H). Only exon-1, exon-2 and exon-5 of the alligator *KissR* gene could be predicted (data not shown).

All predicted KissR proteins from ophidian, chelonian and crocodilian species present the typical seven transmembrane domains (TMD) of the GPCR family (Additional file 4: Figure S3).

#### *No KissR gene in birds*

The TBLASTN analysis performed in bird genomic databases, using previously characterized KissR and newly described sauropsid KissR as queries, only returned genes corresponding to already characterized G-protein coupled receptors different from *KissR* with identity percentages below 40%. This percentage corresponds to the sequence identity shared with the galanin receptor, one of the KissR closest relatives [5]. Thus, we did not find any *KissR* gene candidate in the bird genomic databases.

### Classification of sauropsid KissR

We have recently demonstrated that gnathostome *KissR* can be classified in four different groups, *KissR1*, *KissR2, KissR3* and *KissR*4 [17,21]. We also previously showed that a sauropsid representative, the green anole, presents only the *KissR4*-type gene [21]. In the present study, we have included the newly predicted sauropsid *KissR* in the phylogenetic and syntenic analyses of the gnathostome *KissR* in order to further identify and classify them.

#### *Phylogenetic analysis of KissR genes*

**Figure 6:**
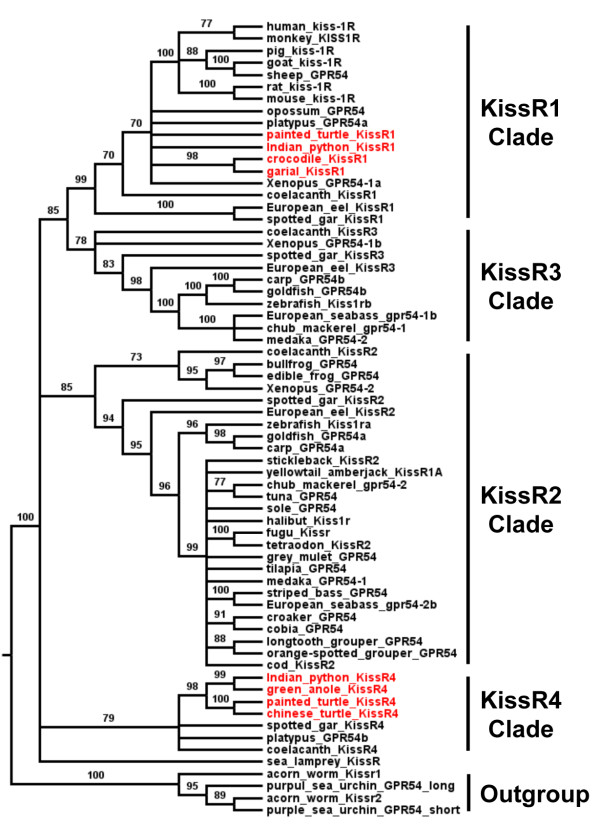
**Consensus phylogenetic tree of vertebrate kisspeptin receptors (KissR).** This phylogenetic tree was constructed based on the amino-acid sequences of KissR using the Maximum Likelihood method with 1,000 bootstrap replicates (for the alignment and references of the sequences see Additional files 5 and 6: Figure S4 and Table S2). The number shown at each branch node indicates the bootstrap value (%); only values and branching above 70% are indicated. The tree was rooted using the two sequences of the hemichordata acorn worm Kissr1 and Kissr2 and the two sequences of echinodermata purple sea urchin GPR54_short and GPR54_long. The sauropsid KissR are in red.

Based on an alignment of 66 KissR peptidic sequences Additional files 5 and 6: Figure S4 and Table S2), and assuming ambulacrarian (acorn worm, *Saccoglossus kowalevskii*, and purple sea urchin, *Strongylocentrotus purpuratus*) KissR as outgroup, a phylogenetic tree was generated that clusters the gnathostome KissR into four clades, which are supported by significant bootstrap values: 99, 85, 78, and 79%, respectively (Figure 6). This is in agreement with our previous study [21].

The KissR1 clade mainly encompasses sarcopterygian KissR1, including mammalian KissR1, Xenopus GPR54-1a, coelacanth KissR1 and four predicted sauropsid sequences, *i.e.* the crocodile, garial, python and painted turtle KissR1. The KissR1 clade also encompasses two actinopterygian KissR1, *i.e.* the spotted gar and European eel KissR1 (Figure 6). These results represent the first evidence for the presence of orthologs to mammalian KissR1 in sauropsids.

The KissR2 clade mainly clusters actinopterygian KissR2, *i.e.* spotted gar KissR2 and most of the previously described teleost KissR. This clade also clusters three sequences from sarcopterygian species, *i.e.* the Xenopus GPR54-2, the bullfrog (*Rana catesbeiana*) GPR54 and the coelacanth KissR2. No sauropsid KissR is present in the KissR2 clade (Figure 6).

The KissR3 clade clusters two sarcopterygian KissR, i.e. the Xenopus GPR54-1b and the coelacanth KissR3, together with actinopterygian KissR, i.e. the spotted gar KissR3 and some teleost KissR including the zebrafish Kiss1rb, the goldfish GPR54b, the medaka GPR54-2 and the European eel KissR3. No sauropsid KissR is present in the KissR3 clade (Figure 6).

The KissR4 clade clusters one actinopterygian KissR, the spotted gar KissR4, with sarcopterygian KissR including four sauropsid sequences, i.e. Chinese turtle, painted turtle, anole and Indian python KissR4 (Figure 6).

This phylogenetic analysis demonstrates that the sauropsid KissR cluster in the KissR1 and/or KissR4 clades, suggesting that only KissR1 and KissR4 are present in the sauropsid lineage.

#### *Syntenic analysis of KissR genes*

In order to test the results obtained with the phylogenetic analysis, and to further understand the evolutionary history of *KissR* genes in sauropsid lineage, we performed a syntenic analysis of the *KissR* neighbouring genes in the same sauropsid genomes as the ones investigated for *Kiss* (section Syntenic analysis). As the synteny analysis of the *KissR* genes has already been investigated and the orthology relationship already demonstrated for human, chicken, green anole, Xenopus, coelacanth and spotted gar [21], we used those species as references in the current study.

**Figure 7:**
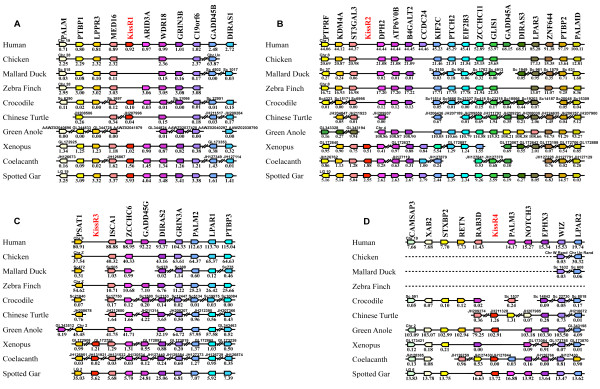
**Conserved genomic synteny of osteichthyan kisspeptin receptors (*KissR*).** Genomic synteny maps comparing the orthologs of *KissR1***(A)**, *KissR2***(B)**, *KissR3***(C)**, *KissR4***(D)** and their neighbouring genes. *KissR* genes are named according to our proposed nomenclature (*KissR1* to *KissR4*, [17,21]). The other genes are named after their human orthologs according to the Human Genome Naming Consortium (HGNC). Orthologs of each gene are shown in the same color. The direction of arrows indicates the gene orientation, with the ID of the genomic segment indicated above and the position of the gene (in 10^-6^ base pairs) indicated below. The full gene names and detailed genomic locations are given in Additional file 8: Table S4.

As for human, Xenopus (*GPR54-1a*), coelacanth and spotted gar *KissR1,* crocodile and Chinese turtle *KissR1* are positioned in genomic regions containing common loci, including *PALM, PTBP1, LPPR3, MED16, ARID3A, WDR18, GRIN3B, C19orf6, GADD45B and DIRAS1* (Figure 7A). The painted turtle, alligator and garial *KissR1* are also located in the vicinity of those genes (data not shown). This supports the orthology relationship of all these *KissR* genes, all considered as *KissR1* genes. This syntenic analysis also supports the absence of chicken, mallard drake, zebra finch and green anole *Kiss1* gene, although the above-mentioned neighbouring genes are present in the respective genomic databases (Figure 7A).

The Xenopus (*GPR54-2*), coelacanth and spotted gar *KissR2* are positioned in genomic regions containing common loci, including *PTPRF, KDM4A, ST3GAL3* and *DPH2* (Figure 7B). As already demonstrated [21], this supports the orthology relationship of these *KissR* genes, considered as *KissR2*. Syntenic analysis also supports the hypothesis that the human, chicken, mallard duck, zebra finch, crocodile, Chinese turtle and green anole genomes do not contain *KissR2* gene, although the above-mentioned neighbouring genes are present in the respective genomic databases (Figure 7B).

The Xenopus (*GPR54-1b*) and spotted gar *KissR3* are positioned in genomic regions containing common loci, including *ISCA* and *ZCCHC6* (Figure 7C). As already demonstrated [21] this supports the orthology relationship of these two *KissR* genes, both considered as *KissR3* genes. The coelacanth *KissR3* is split into scaffolds JH131603.1 and JH131921.1, which are too small to contain any other gene ([21] and Figure 7C). The syntenic analysis also supports the hypothesis that the human, chicken, mallard duck, zebra finch, crocodile, Chinese turtle and green anole genomes do not contain any *KissR3* gene, although the above-mentioned neighbouring genes are present in the respective genomic databases (Figure 7C).

The green anole, coelacanth and spotted gar *KissR4* are positioned in genomic regions containing common loci, including *CAMSAP3, XAB2, STXBP2, RETN, RAB3D, PALM3, NOTCH3, EPHX3, WIZ* and *LPAR2* (Figure 7D), as already shown [21]. The Chinese turtle *KissR4* is located in an isolated scaffold, in the vicinity of the *STXBP2* gene. This supports the orthology relationship of these *KissR* genes, all considered as *KissR4*. This syntenic analysis also supports the absence of human, crocodile, and Xenopus *KissR4* gene, although the above-mentioned neighbouring genes are present in the respective genomic databases (Figure 7D). Syntenic analysis reveals that almost the whole considered region is absent from the bird genomic databases, likely due to incomplete genome sequencing. Indeed, among the eleven considered genes in the *KissR4* syntenic region, only *WIZ* and *LPAR2* genes are present in the chicken and mallard duck genomic databases (Figure 7D).

The results of this syntenic analysis are in agreement with our conclusions based on our phylogenetic analysis, that the sauropsid lineage possesses *KissR1* and *KissR4* genes. These results based on the study of multiple sauropsid genomes, represent the first large-scale investigation and classification of *KissR* genes in sauropsids. We were not able to find any evidence demonstrating the existence of *KissR* gene in the bird genomic databases. However, similarly to what was observed in the syntenic region of *Kiss3* gene (section Syntenic analysis of Kiss genes), our syntenic analysis also emphasizes a drastic lack of genomic information in the bird putative *KissR4* region. In human, this region is also located in the HSA19q. All these analyses lead to the hypothesis that, if birds still possessed a *KissR* gene, it could only be a *KissR4-type* gene. To further investigate the *KissR* existence in birds, we looked for evidence of *KissR* mRNA in the released bird transcriptomic databases.

### Investigation of the *KissR* existence in the released bird transcriptomes

In tetrapods, the *KissR* genes are expressed in a wide range of tissues depending on the species. *Kiss1r* (*KissR1*) transcript is found in placenta, brain, spinal cord, pituitary and pancreas of human [2,3,42], and in rat brain, liver and intestine [18]. In *Xenopus*, the three *KissR* genes are all expressed in the brain. In addition, *GPR54-1a* transcript (*KissR1*) is found in pituitary, testis and intestine, *GPR54-1b* (*KissR3*) in testis and intestine, and *GPR54-2* (*KissR2*) in pituitary and heart [18].

To further investigate the existence of *KissR* in birds, we looked for evidence of *KissR* mRNA in the released bird transcriptomic databases. We performed TBLASTN searches in the same twelve bird transcriptomic databases we used to investigate the potential existence of *Kiss* mRNA). Using the predicted sauropsid KissR peptidic sequences as query, the TBLASTN returned only hits corresponding to other G-protein coupled receptors different from KissR, as already observed when performing a BLAST search on the genomes (see No KissR gene in birds). Thus, we did not find any *KissR* transcript candidate in the bird transcriptomic databases. Although a transcriptomic database cannot totally reflect the gene diversity of a species, the absence of result, in all current databases, is in favour of the loss of *KissR4* in birds.

### Evolutionary scenario of *KissR* gene in the sauropsid lineage

**Figure 8:**
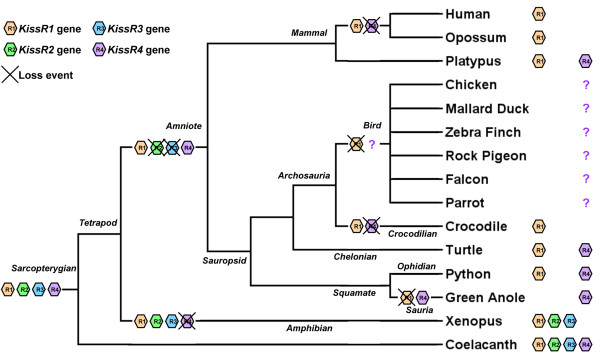
**Current status and proposed evolutionary history of kisspetin receptor (*KissR) *genes among sarcopterygians.** The names of the current representative species of each vertebrate group are given at the end of the final branches, together with the symbol of the *KissR* genes they possess. Due to incomplete bird genome sequencing in the putative region of *KissR4*, the possible existence of *KissR4* in birds cannot be ruled out and is symbolised by a question mark.

Our study revealed the presence of two *KissR* types in sauropsids, *KissR1* and *KissR4*, while the other two types, *KissR2* and *KissR3*, are lacking. Among sarcopterygians, the presence of *KissR2* and *KissR3* could be demonstrated in coelacanth and Xenopus genomes, [18,21] indicating that both genes have been inherited by the sarcopterygian ancestor and more recently by the tetrapod ancestor. As these two genes are lacking in both sauropsid and mammalian lineages, they may have been lost in the amniote ancestor (Figure 8). In contrast, *KissR1* and/or *KissR4* genes are still present in the sauropsid and mammalian lineages (Figure 8), indicating that these two genes were inherited by their common ancestor. Among sauropsids, *KissR1* and *KissR4* genes were inherited by the squamate ancestor, since python still possesses both *KissR1* and *KissR4* genes, while *KissR1* gene would have been lost in lizards (Figure 8). Both *KissR1* and *KissR4* genes were also inherited in the chelonian lineage, as observed in the turtles. The *KissR4* gene would have been lost in the crocodilian lineage since crocodile, alligator and garial only possess *KissR1* (Figure 8). The *KissR1* gene would have been lost in the avian lineage, as shown by synteny analyses (Figure 8). Concerning *KissR4*, the fact that the whole putative *KissR4* genomic region is missing in the current bird genome databases (Figure 7D) does not allow us to assess the absence of this gene in birds by synteny analysis. Thus, even if the absence of *KissR4* in the various genomic and transcriptomic databases is in favour of the loss of this gene, its existence in birds cannot yet be fully excluded (Figure 8).

### Possible physiological significances of *Kiss* loss in birds

Our results provide the first evidence of the presence of a *Kiss2-like* gene in the genomes of three bird species (mallard duck, zebra finch and rock pigeon) and suggest its degeneration and loss of function in the avian lineage. Considering the key-role of Kiss in the control of reproduction in mammals, and the increasing evidences for the conservation of this role in other vertebrate groups such as amphibians and teleosts (for review: [4,12,46-48]), the loss if this regulatory system in birds is specially challenging. Recently in mammals, two other neuropeptides, neurokinin B (NKB, a tachykinin peptide), and dynorphin (DYN, an endogenous opioid peptide), were shown to be co-localized with kisspeptin in a hypothalamic neuron population, named KNDy cells (for Kisspeptin/Neurkinin/Dynorphin) (for review: [49]). These KNDy cells play a major role in steroid feedback and generation of episodic GnRH secretion, as well as in mediating the effects of nutritional status and stress on the reproductive axis. Genes encoding NKB precursor and its receptor (Tacr3) [50], as well as Dyn (*Pdyn: *[31,51]) and its receptor (Kappa opioid receptor, Kor; [52]), have been identified in most vertebrates, including birds. Functional studies in birds would allow to decipher whether those two neuropeptides control GnRH neurons even in the absence of Kisspeptin.

The loss of Kiss function in birds may also reflect the plasticity in the neuromediators and their receptors involved in the control of GnRH neurons and reproductive function throughout vertebrate evolution. For instance, concerning other RF amide neuropeptides, the major inhibitory role of GnIH in reproduction first discovered in birds, is likely less effective in other vertebrate groups (for review: [53]). As another example, large variations in the inhibitory role of dopaminergic neurons in the neuroendocrine control of reproduction have also been observed among vertebrates (for review: [54,55]).

## Conclusion

This study represents the first large-scale investigation of the kisspeptin system in multiple sauropsid genomes, including ophidians, chelonians, crocodilians, and avians. Phylogenetic and syntenic analyses enabled us to classify various predicted sauropsid *Kiss* and *KissR* and to re-construct the evolutionary scenarios of both gene families across the sauropsid radiation. We provide the first evidence for the presence of two *Kiss* genes (*Kiss1* and *Kiss2-types*) and of two *KissR* genes (*KissR1* and *KissR4*-*types*) in the sauropsid lineage. These four genes, also present in the mammalian lineage, would have been inherited from their common amniote ancestor. Among sauropsids, we paid special attention to birds and we demonstrated the existence of a *Kiss2-like* gene in three bird genomes. The divergence of these avian *Kiss2-like* sequences from those of other vertebrates, as well as our inability to find them in the genome of some other birds, reveals *Kiss2* gene degeneration and loss in the avian lineage. These findings represent the first molecular evidence for the existence and fate of a *Kiss gene* in birds.

## Methods

### Genomic databases

The following dedicated genomic databases were investigated from *e*!ENSEMBL genome browser (http://www.ensembl.org/index.html): human, *Homo sapiens*; chicken, *Gallus gallus*; turkey, *Meleagris gallopavo*; zebra finch, *Taeniopygia guttata*; Chinese turtle, *Pelodiscus sinensis*; green anole, *Anolis carolinensis*; Xenopus, *Xenopus tropicalis*; and coelacanth, *Latimeria chalumnae*.

The following dedicated genomic databases were investigated from *Pre*!ENSEMBL genome browser (http://pre.ensembl.org/index.html): collared flycatcher, *Ficedula albicollis*; mallard duck, *Anas platyrhynchos*; budgerigar, *Melopsittacus undulates*; painted turtle, *Chrysemys picta*; and spotted gar, *Lepisosteus oculatus.*

The following dedicated genomic databases were investigated from NCBI genome browser (http://www.ncbi.nlm.nih.gov/genome/browse/): medium ground finch, *Geospiza fortis*; rock pigeon, *Columba livia*; saker falcon, *Falco cherrug*; peregrine falcon, *Falco peregrinus*; Tibetan ground-tit, *Pseudopodoces humilis*; Puerto-Rican parrot, *Amazona vittata*; and Indian python, *Python molurus*.

The following dedicated genomic databases were investigated from croc genomes web site (http://crocgenomes.org/): Alligator, *Alligator mississippiensis*; garial, *Gavialis gangeticus*; and crocodile, *Crocodylus porosus*.

### Transcriptomic databases

The following bird transcriptomic databases were investigated from NCBI Trace browser (http://www.ncbi.nlm.nih.gov/Traces/sra/?view=run_browser): pied flycatcher, *Ficedula hypoleuca* (SRX012275); zebra finch (SRP003283); ring-necked dove, *Streptopelia roseogrisea* (SRX012364); blue tit, *Parus caeruleus* (SRX012276); European crow, *Corvus corone* (SRX006755 and SRX006756); American crow, *Corvus brachyrhynchos* (SRX012416); ruby-throated hummingbird, *Archilochus colubris* (SRX012361); Anna’s hummingbird, *Calypte anna* (SRX012362); golden collared manakin, *Manacus vitellinus* (SRX012420); emu, *Dromaius novaehollandiae* (SRX012419); and budgerigar, *Melopsittacus undulatus* (SRX012363).

The chicken transcriptomic databases were investigated from the archive at ftp://www.chick.manchester.ac.uk/pub/chickest/.

### Gene predictions

#### *TBLASTN search*

The TBLASTN algorithm (search sensitivity: near exact match short) of the *e*!ENSEMBL website (http://www.ensembl.org/index.html) was used on the genomic databases available at the *e*!ENSEMBL or *pre*!ENSEMBL website. The TBLASTN algorithm of the CLC DNA Workbench software (CLC bio, Aarhus, Denmark) was used on the other investigated genomic and transcriptomic databases.

#### *Kiss genes*

In mammals, the *Kiss1* gene is composed of three exons and two introns as shown in human [56], pig (*Sus scrofa*) [57], and mouse (*Mus musculus*) [58]. The first exon only codes for 5′ un-translated region (UTR), while the coding sequence (CDS) is split into the two other exons. The sequence encoding the mature peptides, including the largely conserved Kp(10) sequence, is contained in the last of these two exons. In the other vertebrates, the *Kiss* CDS is also split into two exons, with the mature peptides, including Kp(10), encoded by the last exon (for review: [19]). In all vertebrate species, the Kp(10) sequences are directly followed in C-terminal end by the sequence, “G-Basic-Basic” or “G-Basic-Stop”, characteristic of the conserved proteolytic cleavage and alpha-amidation sites of neuropeptides [32]. Considering that the *Kiss* gene sequences are highly variable among species except for the sequence encoding Kp(10) and the flanking proteolytic cleavage and alpha-amidation site, we focused our predictions on the genomic ORF encoding these sequences.

The peptidic sequences of *Xenopus Kiss1a*, *Kiss1b* and *Kiss2* (*Kiss1, Kiss3* and *Kiss2* in this study, respectively) [18], coelacanth *Kiss1*, *Kiss2* and *Kiss3,* and elephant shark *Kiss1*, *Kiss2* and *Kiss3*[17] were used as query in TBLASTN search to identify the ORF encoding Kp(10) in the various investigated genomic and transcriptomic databases. We applied ORF-finder tool of the CLC DNA Workbench software to retrieve ORF encompassing Kp(10). Novel sauropsid Kiss sequences, predicted in the present study, were used as query to blast again bird genomic and transcriptomic data bases.

For the bird *Kiss-like* ORFs, the potential splice acceptor sites (T/C rich regions followed by an AG motif), located between the stop codon and the region encoding the Kp(10), were predicted using HSF (Human splicing Finder) v2.4.1 [59]. Only splice acceptor sites predicted with a consensus value above 80 were retained as potential candidates in this study.

#### *KissR genes*

The exon-intron structure of the *KissR* genes is well conserved among vertebrates and it is made of five exons and four introns (for review: [19]). To retrieve the CDS of new *KissR* genes from the various investigated databases, the TBLASTN searches were performed using the human Kiss1r (KissR1 in this study), the three xenopus KissR, the four coelacanth KissR and the green anole KissR4 peptidic sequences as query. The splice junctions were predicted using the HSF (Human splicing Finder) v2.4.1 [59] and manually checked using the empirical nucleotidic splicing signatures, *i.e.* intron begins with “GT” and ends with “AG” [21]. Novel sauropsid KissR sequences, predicted in the present study, were used as query to blast again bird genomic and transcriptomic data bases.

### Prediction of peptidic features

Putative mature Kiss-peptides of various lengths were deduced after the prediction of proteolytic cleavage site from the translated ORF encompassing Kp(10). These putative proteolytic cleavage sites were predicted using NeuroPred tool [60].

The 7 transmembrane domains of the predicted KissR were determined using TMHMM software (TMHMM Server v. 2.0).

### Phylogenetic analysis

#### *Kiss phylogeny*

68 sequences, from 33a.a. to 65a.a., and each one composed of a predicted mature Kisspeptin followed by its proteolytic cleavage and alpha-amidation site, were first aligned using ClustalW [61], then manually adjusted. The JTT (Jones, Taylor and Thornton) protein substitution matrix of the resulting alignment was determined using ProTest software [62]. Phylogenetic analysis of the predicted mature Kisspeptin alignment was performed using the neighbour joining method (MEGA 5.1 software), with 1,000 bootstrap replicates.

#### *KissR phylogeny*

Amino-acid sequences of 66 known or predicted *KissR* were first aligned using ClustalW [61], then manually adjusted. The JTT (Jones, Taylor and Thornton) protein substitution matrix of the resulting alignment was determined using ProTest software [62]. Phylogenetic analysis of the KissR sequence alignment was performed using the maximum likelihood method (RaxML software [63]), with 1,000 bootstrap replicates.

### Syntenic analyses

Synteny maps of the conserved genomic regions in human, chicken, zebra finch, Chinese turtle, green anole, *Xenopus,* coelacanth and spotted gar were performed using the PhyloView of Genomicus v71.01 web site (http://www.genomicus.biologie.ens.fr/genomicus-71.01/cgi-bin/search.pl) [64]. The analysis of the neighbouring genes of the four coelacanth *KissR* paralogs was completed by manual gene annotation of the coelacanth genome. The analyses of the mallard duck and painted turtle genomic regions were performed using the preliminary gene annotations of the genome assembly duck1 and ChrPicBel3.0.1 generated by Ensembl release 71. The synteny analyses of rock pigeon, alligator, garial and crocodile conserved genomic regions were obtained performing TBLASTN (CLC DNA Workbench 6 software) searches in the corresponding genomic databases and for all the conserved genes of the *Kiss* and *KissR* syntenies. For each of those genes, the peptidic sequences of human, green anole and chicken orthologs were used as query, as far as they were referenced in databases.

### Partial cloning of the mallard duck *Kiss2-*like gene

Genomic DNA from an adult mallard hen liver, provided by the INRA-Nouzilly (France), was extracted using DNeasy Blood & Tissue Kit (Qiagen, Hilden, Germany). 5′ and 3′ surrounding regions of the predicted ORF encoding mallard duck Kiss2-like were used to design specific forward (5′-GCTGCAAGGGAACAACATTC-3′) and reverse (5′-CAGTCTAATACCCAGCACCAGTC-3′) primers in order to amplify it by PCR. Classical PCRs were performed as follows: an initial step of polymerase activation for 3 min at 94°C; then 35 cycles with 30 s at 94°C for denaturing, 30 s at 60 for annealing, 1 min 30 s at 72°C for primer extension, and a single final extension step of 5 min at 72°C. PCR products were purified with the QUIAquick PCR Purification Kit (Qiagen, Hilden, Germany) and inserted in a PCR™4-TOPO® TA vector provided by the TOPO® TA Cloning® Kit (Invitrogen). The vectors were then transfected in One Shot® TOP10 Chemically Competent E. coli (Invitrogen). After the bacteria containing a vector with insert had grown in miniprep cultures, vectors were extracted and purified using QUIAquick Spin Miniprep Kit (Qiagen, Hilden, Germany). Their inserts were then sequenced at GATC biotech Ltd (Konstanz, Germany). The obtained sequence was submitted to EMBL under the accession number: HG328246.

### Sequence availability

The partial genomic sequence of mallard duck Kiss2-like (exon 2), which has been cloned, has been submitted to EMBL under the accession number HG328246 (http://www.ebi.ac.uk/ena/data/view/HG328246).

## Availability of supporting data

The predicted sauropsid Kiss and KissR sequences are available in Additional files 1 and 4, respectively (Figure S1 and S3). The alignment matrices of vertebrate Kiss and KissR proteins are available in Additional files 2 and 5, respectively (Figure S2 and S4) The references of the sequences of the Kiss and KissR used in the phylogeny analyses are provided in Additional files 3 and 6, respectively (Tables S1 and S2). The coordinates of the genes displayed in the Kiss and KissR syntenies are available in Additional files 7 and 8, respectively (Tables S3 and S4).

## Competing interests

The authors declare that they have no competing interests.

## Authors’ contributions

Conceived and designed the study: JP, BQ, SD. Performed the lab work: JP. Contributed materials/ tools: SD, PC. Analyzed the data: JP, AGL, BQ, SD. Wrote the paper: JP, AGL, SD. Provided comments on the manuscript: KR, BQ, PC. All authors read and approved the final manuscript.

## Supplementary Material

Additional file 1: Figure S1 Predicted sauropsid Kiss ORFs. Nucleotide and deduced amino-acid sequences of the genomic region of the predicted sauropsid Kiss open reading frames (ORF). Nucleotides (top) are numbered from 5′ to 3′. The amino-acid residues (bottom) are numbered beginning with the first residue in the ORF. The asterisks (*) indicate the stop codons delineating the ORF. The predicted Kp(10) peptides are shaded in grey and the C-terminal predicted proteolytic and alpha-amidation sites are shaded in black. The predicted putative splice acceptor sites (AG) located between the stop codon and the region encoding the rock pigeon Kp(10) are coloured in red, and the preceding T/C rich sequences are underlined. No such sites could be predicted in the zebra finch. Click here for file

Additional file 2: Figure S2 Alignment of the amino-acid sequences of 68 long mature kisspeptins used for the phylogenetic analysis (Figure 3). The amino-acid sequences were aligned by ClustalW and manually adjusted. The amino-acid sharing similar physico-chemical properties are represented with the same color. The references of the sequences are provided in the Additional file 3: Table S1. Click here for file

Additional file 3: Table S1 References of the sequences used in the Kiss phylogeny analysis (Figure 4). Click here for file

Additional file 4: Figure S3 Predicted sauropsid KissR CDS. Nucleotide and deduced amino-acid sequences of the predicted sauropsid KissR coding DNA sequences (CDS). Nucleotides (top) are numbered from 5′ to 3′. The amino-acid residues (bottom) are numbered beginning with the first residue in the ORF. The asterisks (*) indicate the stop codons. The nucleotides at the exon-exon junctions are in red. The transmembrane domains (TMD) are underlined and numbered according to their position from N-terminal to C-terminal ends of the receptor. Click here for file

Additional file 5: Figure S4 Alignment of the amino-acid sequences of 66 kisspeptin receptors used for the phylogenetic analysis (Figure 6). The amino-acid sequences were aligned by ClustalW and manually adjusted. The amino-acid sharing similar physico-chemical properties are represented with the same color. The references of the sequences are provided in the Additional file 6: Table S2. Click here for file

Additional file 6: Table S2 References of the sequences used in the KissR phylogeny analysis (Figure 7) [65]. Click here for file

Additional file 7: Table S3 Names, references and locations of the genes used in the synteny analysis of the *Kiss* genes (Figure 4). Click here for file

Additional file 8: Table S4 Names, references and locations of the genes used in the synteny analysis of the kisspeptin receptors *(KissR)* genes (Figure 7). Click here for file
