# Supplementary material for: Looking for the bird Kiss: evolutionary scenario in sauropsids
Source: BMC Evol Biol. 2014 Feb 19;14:30. doi: 10.1186/1471-2148-14-30 (PMC4015844; doi:10.1186/1471-2148-14-30)
Supplement: Additional file 1: Figure S1 — Predicted sauropsid Kiss ORFs. Nucleotide and deduced amino-acid sequences of the genomic region of the predicted sauropsid Kiss open reading frames (ORF). Nucleotides (top) are numbered from 5′ to 3′. The amino-acid residues (bottom) are numbered beginning with the first residue in the ORF. The asterisks (*) indicate the stop codons delineating the ORF. The predicted Kp(10) peptides are shaded in grey and the C-terminal predicted proteolytic and alpha-amidation sites are shaded in black. The predicted putative splice acceptor sites (AG) located between the stop codon and the region encoding the rock pigeon Kp(10) are coloured in red, and the preceding T/C rich sequences are underlined. No such sites could be predicted in the zebra finch. [file 1471-2148-14-30-S1.pdf]

>Indian python ORF Kiss1 |Contig 25636949|

1 - TAAAAGTTATTTTACATGCTTTGTCCTTCCCCCACGCTGATATGTTCTCTCTATAAACATTTCTCA - 66
1 - * K L F Y M L C P S P T L I C S L Y K H F S - 22

67 - TTCTTTCTAGCTAAACCTCCCAGAGGCATTGCCAATTGGGTCCACTGGGCTGATGACATCCCATGT - 132
23 - F F L A K P P R G I A N W V H W A D D I P C - 44

133 - TCTAAAAGGAGCTTCATCTCTGGCAAAGAAGGTCTGAAATCCACGGTAACCCTTCTGTGCAAGCGG - 198
45 - S K R S F I S G K E G L K S T V T L L C K R - 66

199 - CAAGAGCATCAGCTGCAGTTATGGCCAGGGACATCTCCCACAAGAAACAAGGGGGTCCCATTGCTG - 264
67 - Q E H Q L Q L W P G T S P T R N K G V P L L - 88

265 - CAAGAGGCATTGCTGTTAGAAGGGGACCATGATCTCTCCACCTACAACTTGAATTCCTTTGGGTTA - 330
89 - Q E A L L L E G D H D L S T **Y N L N S F G L** - 110

331 - AGATATGGCAAGAGGCAAGCAGTGAAAGCCCAGGAGAAAATATGA - 375
111 - **R Y** **G K R** Q A V K A Q E K I * - 125

>Indian python ORF Kiss2 |Contig 26442132|

1 - TAGTGTCATCCATTTAAGGCTGCACCAGTTGGTTTGTCCCAGTGTTTCATGGTTCCTTGCCATATC - 66
1 - * C H P F K A A P V G L S Q C F M V P C H I - 22

67 - CCACATTCTGCAACTGAACCTCTTTTCCTAGATCTACCTCCTGAATGGCAAAAAAAGAGGAACTCC - 132
23 - P H S A T E P L F L D L P P E W Q K K R N S - 44

133 - TATTACGCAACCCAAGACTCTGAGGATCCCAACGGGGCCGATCCCACCAGTCTTTGCTACTTTATC - 198
45 - Y Y A T Q D S E D P N G A D P T S L C Y F I - 66

199 - CAAGAGAGTGAGACAGAAAGTCAGATATCGTGTAGGCTCCGGTTTACCAGAAGTAGATTTAACTTC - 264
67 - Q E S E T E S Q I S C R L R F T R S R **F N F** - 88

265 - AATTCTTTCGGACTCCGTTTCGGGAAACGGCAGGGGTCTCCCTTGGCTGATGATGGACAATTGGGT - 330
89 - **N S F G L R F** **G K R** Q G S P L A D D G Q L G - 110

331 - TCTCGAAACAGTGGGAATACCATGGAAGTCCTGCCAAAGTCCAACCTGAAGAGGAGATTGGCTTAG - 396
111 - S R N S G N T M E V L P K S N L K R R L A * - 132

>Painted turtle ORF Kiss1 |Scaffold JH584856|

```
  1 - TAAGATTATGGCAGGCCCTGTGAATTCTTTACTATTTATTTGTTTGGATTGCGGTGGTGCATAGGA -  66
  1 - *  D  Y  G  R  P  C  E  F  F  T  I  Y  L  F  G  L  R  W  C  I  G   -  22

 67 - GCTTCAGCCACGCACCAGGACCTCATGTGCTGGGTGCAGTGCAAACACAGAACAAAAAGACAGACT - 132
 23 - A  S  A  T  H  Q  D  L  M  C  W  V  Q  C  K  H  R  T  K  R  Q  T   -  44

133 - CTGCCCCAAAGACACTGCCTGCCACTTGCCACTGGTGAGTCTGACACTGACCACTTTCCCTCCTCA - 198
 45 - L  P  Q  R  H  C  L  P  L  A  T  G  E  S  D  T  D  H  F  P  S  S   -  66

199 - ACTCCTTCTGCGCCTTTCTCTGTAGGTGACCATTTCAAATACCTGGCCAGCCCGGCCCACTGGGAT - 264
 67 - T  P  S  A  P  F  S  V  G  D  H  F  K  Y  L  A  S  P  A  H  W  D   -  88

265 - CAAGCCATTCCTTGCTCGGAAAGCAAGCCCAGCCCTGGGAAGGCAGAACCAAGATCCACGCCACCC - 330
 89 - Q  A  I  P  C  S  E  S  K  P  S  P  G  K  A  E  P  R  S  T  P  P   - 110

331 - CTGCTGTGCAAGCCGCAGGAGGACAGCCAGGTCCAGCTGGGGCAAGGAATCCATCCGGCCAGGAGC - 396
111 - L  L  C  K  P  Q  E  D  S  Q  V  Q  L  G  Q  G  I  H  P  A  R  S   - 132

397 - AGAGCCATCTCTGTCCCCCAGGGCTCGCTGCTCGTGGAGCGGGAGAAGGATCTCTCCGCCTACAAC - 462
133 - R  A  I  S  V  P  Q  G  S  L  L  V  E  R  E  K  D  L  S  A  Y  N   - 154

463 - TGGAACTCGTTCGGCTTGAGATACGGCAAGAGGCAAGCAGACACCAGGGAAGCCAAGGTGAAAATA - 528
155 - W  N  S  F  G  L  R  Y  G  K  R  Q  A  D  T  R  E  A  K  V  K  I   - 176

529 - TTGTGA                                                             - 534
177 - L  *                                                              - 178
```

>Painted turtle ORF Kiss2 |Scaffold JH584680|

```
  1 - TAAACCTACTGCATGAGATTCCTAAGGGAGGTGCTTGCAAGGTGTGTGCATGTGGAAGACCTTGAG -  66
  1 - *  T  Y  C  M  R  F  L  R  E  V  L  A  R  C  V  H  V  E  D  L  E   -  22

 67 - TTTTGCACACTCTTTCAAGGTATTTTGGATTTCCTCAGTGGTGATGGTGGTGTGTGTTTTGCCCTA - 132
 23 - F  C  T  L  F  Q  G  I  L  D  F  L  S  G  D  G  G  V  C  F  A  L   -  44

133 - GATGCTGAATATCCAGTCAGTGACATACAAGCCAAGAGGAACTCCTATCAGGCGAGCCAAGATGCC - 198
 45 - D  A  E  Y  P  V  S  D  I  Q  A  K  R  N  S  Y  Q  A  S  Q  D  A   -  66

199 - GATAGCCCCAATTCTGCAGAGCAATCCAGCCTCTGCTACTTTATCCAGGAGAGTGAGATTGCGAGT - 264
 67 - D  S  P  N  S  A  E  Q  S  S  L  C  Y  F  I  Q  E  S  E  I  A  S   -  88

265 - CAGATCTCCTGCAGACTGCGATTCACCAGGAGCAAGTTTAATTTTAACCCTTTTGGACTTCGGTTT - 330
 89 - Q  I  S  C  R  L  R  F  T  R  S  K  F  N  F  N  P  F  G  L  R  F   - 110

331 - GGGAAAAGGCAACAAGTCAGCTTGGCCAGCAAAAGGGATCCAATCACTTTAAGCAGTGTCAAAAAA - 396
111 - G  K  R  Q  Q  V  S  L  A  S  K  R  D  P  I  T  L  S  S  V  K  K   - 132

397 - TTACCATCCCTATTAAAGTTCAAACTAAACCAAATGGTGCCCTGGTGTGGAGACTTTGGGGAGCAG - 462
133 - L  P  S  L  L  K  F  K  L  N  Q  M  V  P  W  C  G  D  F  G  E  Q   - 154

463 - GATTGTTAA                                                          - 471
155 - D  C  *                                                           - 157
```

>Chinese turtle ORF Kiss1 |Scaffold JH211545|

1 - TAAGATTATGGCAGGCCCTGTGAATTCTTTACTATTTATTTGTTTGGATTGCGGTGGTGCATAGGA - 66
1 - * D Y G R P C E F F T I Y L F G L R W C I G - 22

67 - GCTTCAGCCACGCACCAGGACCTCATGTGCTGGGTGCAGTGCAAACACAGAACAAAAAGACAGACT - 132
23 - A S A T H Q D L M C W V Q C K H R T K R Q T - 44

133 - CTGCCCCAAAGACACTGCCTGCCACTTGCCACTGGTGAGTCTGACACTGACCACTTTCCCTCCTCA - 198
45 - L P Q R H C L P L A T G E S D T D H F P S S - 66

199 - ACTCCTTCTGCGCCTTTCTCTGTAGGTGACCATTTCAAATACCTGGCCAGCCCGGCCCACTGGGAT - 264
67 - T P S A P F S V G D H F K Y L A S P A H W D - 88

265 - CAAGCCATTCCTTGCTCGGAAAGCAAGCCCAGCCCTGGGAAGGCAGAACCAAGATCCACGCCACCC - 330
89 - Q A I P C S E S K P S P G K A E P R S T P P - 110

331 - CTGCTGTGCAAGCCGCAGGAGGACAGCCAGGTCCAGCTGGGGCAAGGAATCCATCCGGCCAGGAGC - 396
111 - L L C K P Q E D S Q V Q L G Q G I H P A R S - 132

397 - AGAGCCATCTCTGTCCCCCAGGGCTCGCTGCTCGTGGAGCGGGAGAAGGATCTCTCCGCCTACAAC - 462
133 - R A I S V P Q G S L L V E R E K D L S A **Y N** - 154

463 - TGGAACTCGTTCGGCTTGAGATACGGCAAGAGGCAAGCAGACACCAGGGAAGCCAAGGTGAAAATA - 528
155 - **W N S F G L R Y** **G K R** Q A D T R E A K V K I - 176

529 - TTGTGA - 534
177 - L * - 178

>Chinese turtle ORF Kiss2 |Scaffold JH209943|

1 - TAAACCTACTGCATGAGATTCCTAAGGGAGGTGCTTGCAAGGTGTGTGCATGTGGAAGACCTTGAG - 66
1 - * T Y C M R F L R E V L A R C V H V E D L E - 22

67 - TTTTGCACACTCTTTCAAGGTATTTTGGATTTCCTCAGTGGTGATGGTGGTGTGTGTTTTGCCCTA - 132
23 - F C T L F Q G I L D F L S G D G G V C F A L - 44

133 - GATGCTGAATATCCAGTCAGTGACATACAAGCCAAGAGGAACTCCTATCAGGCGAGCCAAGATGCC - 198
45 - D A E Y P V S D I Q A K R N S Y Q A S Q D A - 66

199 - GATAGCCCCAATTCTGCAGAGCAATCCAGCCTCTGCTACTTTATCCAGGAGAGTGAGATTGCGAGT - 264
67 - D S P N S A E Q S S L C Y F I Q E S E I A S - 88

265 - CAGATCTCCTGCAGACTGCGATTCACCAGGAGCAAGTTTAATTTTAACCCTTTTGGACTTCGGTTT - 330
89 - Q I S C R L R F T R S K **F N F N P F G L R F** - 110

331 - GGGAAAAGGCAACAAGTCAGCTTGGCCAGCAAAAGGGATCCAATCACTTTAAGCAGTGTCAAAAAA - 396
111 - **G K R** Q Q V S L A S K R D P I T L S S V K K - 132

397 - TTACCATCCCTATTAAAGTTCAAACTAAACCAAATGGTGCCCTGGTGTGGAGACTTTGGGGAGCAG - 462
133 - L P S L L K F K L N Q M V P W C G D F G E Q - 154

463 - GATTGTTAA - 471
155 - D C * - 157

>Saltwater crocodile ORF Kiss1 |Scaffold 14567|

```
  1 - TAAAAGATATCGCTTCTACCCAAAGAAGCTTTGCCTATGTCCTTAGACCAACATGGCTACAACCAA -  66
  1 - *  K  I  S  L  L  P  K  E  A  L  P  M  S  L  D  Q  H  G  Y  N  Q   -  22

 67 - AAACCCTGCATCTACGGTGAGGCCCGTTCCCTTCTTAACGCTCCCTCTCTCCCTTTCGCTGTAGGT - 132
 23 - K  P  C  I  Y  G  E  A  R  S  L  L  N  A  P  S  L  P  F  A  V  G   -  44

133 - GTCCATTCCAACCCCACTCATTGCTTGGAGAGAAAGCCCAGCCCTGGGAAGACAGAGCTGAAATCC - 198
 45 - V  H  S  N  P  T  H  C  L  E  R  K  P  S  P  G  K  T  E  L  K  S   -  66

199 - ATGCCAGCTGTCCCATGCCGGCGCCGGGGGAGCCCAGCCCAGCTGTGGGAAGGCATCTCTCTGTTC - 264
 67 - M  P  A  V  P  C  R  R  R  G  S  P  A  Q  L  W  E  G  I  S  L  F   -  88

265 - CGGAGAAGAGGGAATGCTGCACCTCAGAGGTCGCTGCTGGTGGACCGGGAGAAGGATCTCTCCACC - 330
 89 - R  R  R  G  N  A  A  P  Q  R  S  L  L  V  D  R  E  K  D  L  S  T   - 110

331 - TACAACTGGAACTCATTTGGCCTGCGATACGGCAAGAGGCAAGCAGGCGTGGAGGAAGCCAATGTG - 396
111 - Y  N  W  N  S  F  G  L  R  Y  G  K  R  Q  A  G  V  E  E  A  N  V   - 132

397 - AAAATATGGTGA                                                       - 408
133 - K  I  W  *                                                         - 136
```

>American alligator ORF Kiss1 |Scaffold 4128|

1 - TAAGCTTGGGGCCGTTCATTCACACAGCAAAGAGATGGCCCTACCTCAGAGGTCCTGCCCAGTGCT - 66
1 - * A W G R S F T Q Q R D G P T S E V L P S A - 22

67 - CATTTGCTCATCTCAGAAGAGCAGAGACTCCCTGAAGAAGGGTGCTTGTGCCCAAAAGCTTGCAGA - 132
23 - H L L I S E E Q R L P E E G C L C P K A C R - 44

133 - GAACTTTTTCCCAACTACTCAGTTGGTCTAATAAAAGATATCACATCTACCCAAAGAAGCTTGCCT - 198
45 - E L F P N Y S V G L I K D I T S T Q R S L P - 66

199 - GCCTTGTCCTTAGACCAACATGGCTACAACCAAAAACCCTACATCTACGGTGAAGACCTTTCTCTT - 264
67 - A L S L D Q H G Y N Q K P Y I Y G E D L S L - 88

265 - CTTAATGCTCCCTCTCTCCCTTTCGCTGTAGACATCCATTCCAACCCCACTCATTGCTCGGAGAGA - 330
89 - L N A P S L P F A V D I H S N P T H C S E R - 110

331 - AAGCCCAGCCCTGGGAAGACAGAGCTGAAATCTATGCCAGCCCTCCCATGCCGGCGCCGGGGGAGC - 396
111 - K P S P G K T E L K S M P A L P C R R R G S - 132

397 - CCGGCCCAGCTGTGGGAAGGGATCTCTCTCTTCCGGAGAAGAGGGAATGCTGCACCTCAGAGGTCG - 462
133 - P A Q L W E G I S L F R R R G N A A P Q R S - 154

463 - CTGCTGGTGGACCGGGAGAAGGATCTCTCCACCTACAACTGGAACTCATTTGGCCTACGATACGGC - 528
155 - L L V D R E K D L S T **Y N W N S F G L R Y** **G** - 176

529 - AAGAGGCAAGCAGGCGTTGAGGAAGCCAACGTGAAAATATGGTGA - 573
177 - **K R** Q A G V E E A N V K I W * - 191

>Indian garial ORF Kiss1 |Scaffold 21193|

```
  1 - TAGACCAACCAAAAACCCTGCATCTATGGTGAGGCCCTTTCCCTTCTTAACGCTCCCTCTCTCCCT -  66
  1 - *  T  N  Q  K  P  C  I  Y  G  E  A  L  S  L  L  N  A  P  S  L  P   -  22

 67 - TTCGCTGTAGGCGTCCATTCCAACCCCACTCATTGCTTGGAGAGAAAGCCCAGCTCTGGGAAGACA - 132
 23 - F  A  V  G  V  H  S  N  P  T  H  C  L  E  R  K  P  S  S  G  K  T   -  44

133 - GAGCTGAAATCCATGCCAGCCGTCCCATGCTGGCGCCGGGGGCGCCCAGCCCAGCTGTGGGAAGGG - 198
 45 - E  L  K  S  M  P  A  V  P  C  W  R  R  G  R  P  A  Q  L  W  E  G   -  66

199 - ATCTCTCTGTTCCGGAGAAGAGGCAATGCTGCACCTCAGAGGTCACTGCTGGTGGACCGGGAGAAG - 264
 67 - I  S  L  F  R  R  R  G  N  A  A  P  Q  R  S  L  L  V  D  R  E  K   -  88

265 - GATCTCTCCACCTACAACTGGAACTCATTTGGCCTGCGATACGGCAAGAGGCAAGCAGGCGTGGAG - 330
 89 - D  L  S  T  Y  N  W  N  S  F  G  L  R  Y  G  K  R  Q  A  G  V  E   - 110

331 - GAAGCCAACGTGAAAATATGGTGA                                           - 354
111 - E  A  N  V  K  I  W  *                                             - 132
```

>Rock pigeon ORF Kiss2-like |Contig 42221|

```
 1 - TAGCCATCCTGCAGATATCAGCTCACCAGAGGCAGGTTTAATGTTAATCCTTTGGGAGTTGGATTT -  66
 1 - *  P  S  C  R  Y  Q  L  T  R  G  R  F  N  V  N  P  L  G  V  G  F    -  22

67 - GGAAAAGGATGCTGGGGTAGCCTGGCCACTAAGATGGATCCTGCAACTTAG                - 117
23 - G  K  G  C  W  G  S  L  A  T  K  M  D  P  A  T  *                   -  39
```

>Zebra finch ORF Kiss2-like |Chromosome 1A|

```
  1 - TAACAGCTTGCCATGGGCAAGTTTAAGGTTAATCTTCTTGGACTTGGCTTTGGAAAATTATGCCAG -  66
  1 - *  Q  L  A  M  G  K  F  K  V  N  L  L  G  L  G  F  G  K  L  C  Q  -  22

 67 - AGTAGCTTGGCCACCCAGATGGATTCTGCAACTCAGATAAGCATCAGAATGCCACCGTCCTATTTA - 132
 23 - S  S  L  A  T  Q  M  D  S  A  T  Q  I  S  I  R  M  P  P  S  Y  L  -  44

133 - AAGTTTAAAATAAATGCTGTGATGAGCAGGTATCCCTCTGAGGGACTGGACTGCTCAGTGCATAAT - 198
 45 - K  F  K  I  N  A  V  M  S  R  Y  P  S  E  G  L  D  C  S  V  H  N  -  66

199 - CTATGCTACAATTCACGTGTGCCATTTTCCCATGTGAGCAGAGATTTGGGAACATTCCCTTTTGAA - 264
 67 - L  C  Y  N  S  R  V  P  F  S  H  V  S  R  D  L  G  T  F  P  F  E  -  88

265 - GCACCTCCCCCAGGCACCTGA                                              - 285
 89 - A  P  P  P  G  T  *                                                -  95
```
