# Supplementary figures and images for: Looking for the bird Kiss: evolutionary scenario in sauropsids
Source: BMC Evol Biol. 2014 Feb 19;14:30. doi: 10.1186/1471-2148-14-30 (PMC4015844; doi:10.1186/1471-2148-14-30)

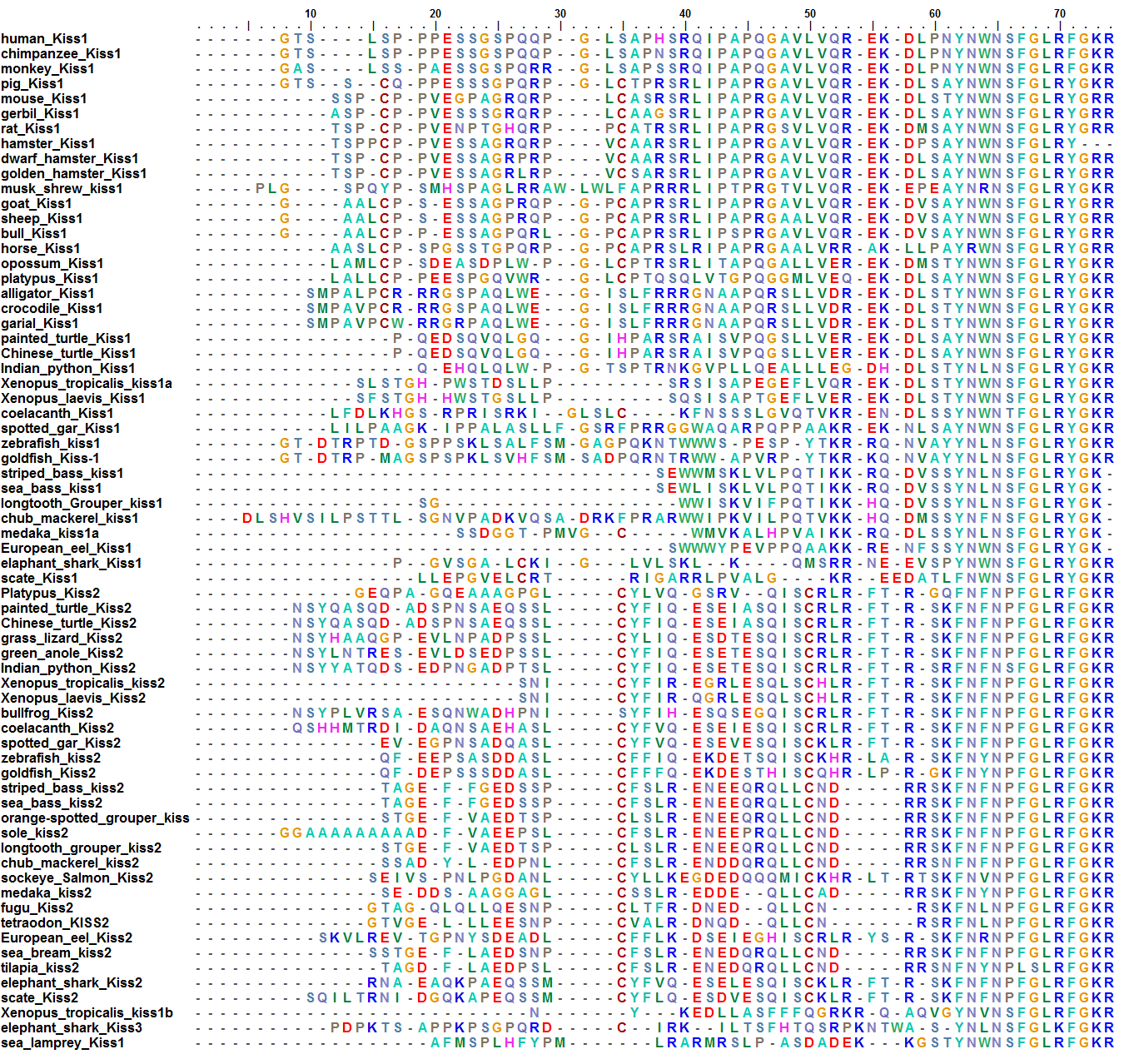

Supplement: Additional file 2: Figure S2 — Alignment of the amino-acid sequences of 68 long mature kisspeptins used for the phylogenetic analysis (Figure 3). The amino-acid sequences were aligned by ClustalW and manually adjusted. The amino-acid sharing similar physico-chemical properties are represented with the same color. The references of the sequences are provided in the Additional file 3: Table S1. [file 1471-2148-14-30-S2.tiff]

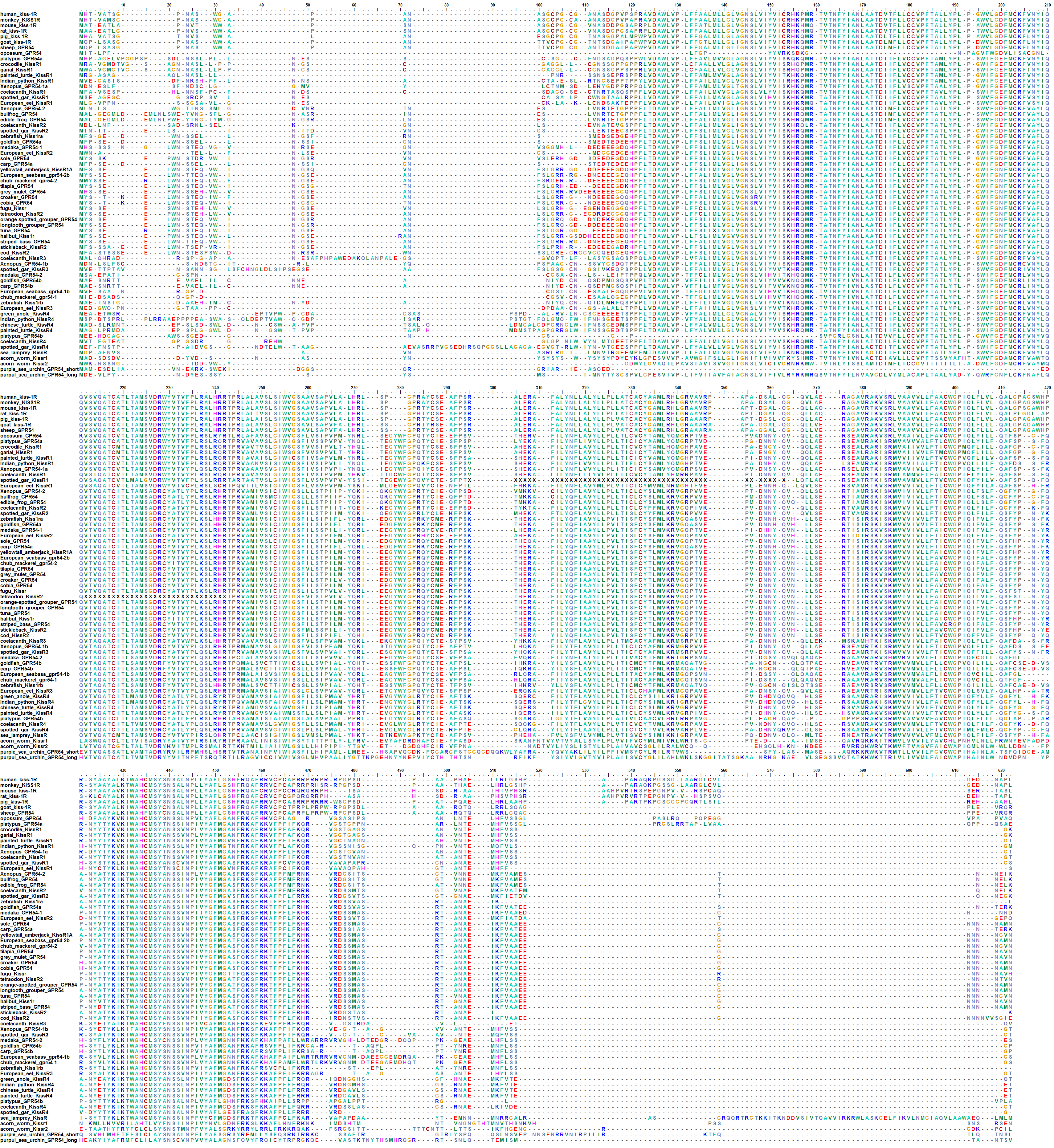

Supplement: Additional file 5: Figure S4 — Alignment of the amino-acid sequences of 66 kisspeptin receptors used for the phylogenetic analysis (Figure 6). The amino-acid sequences were aligned by ClustalW and manually adjusted. The amino-acid sharing similar physico-chemical properties are represented with the same color. The references of the sequences are provided in the Additional file 6: Table S2. [file 1471-2148-14-30-S5.tiff]
