# Supplementary material for: Looking for the bird Kiss: evolutionary scenario in sauropsids
Source: BMC Evol Biol. 2014 Feb 19;14:30. doi: 10.1186/1471-2148-14-30 (PMC4015844; doi:10.1186/1471-2148-14-30)
Supplement: Additional file 4: Figure S3 — Predicted sauropsid KissR CDS. Nucleotide and deduced amino-acid sequences of the predicted sauropsid KissR coding DNA sequences (CDS). Nucleotides (top) are numbered from 5′ to 3′. The amino-acid residues (bottom) are numbered beginning with the first residue in the ORF. The asterisks (*) indicate the stop codons. The nucleotides at the exon-exon junctions are in red. The transmembrane domains (TMD) are underlined and numbered according to their position from N-terminal to C-terminal ends of the receptor. [file 1471-2148-14-30-S4.pdf]

Fig. S3A

>Indian python KissR1

```
   1 - ATG GTA GAG GGA GCA TCC ATC TCT GAT TTC AAC AAG TCC CAC TTC TTT CTG AAC AAT TCA GCC  -   63
   1 -  M   V   E   G   A   S   I   S   D   F   N   K   S   H   F   F   L   N   N   S   A   -   21

  64 - TGC ACT GCA GAG TCG TTG CGC ACT AAT GGC TCT GAG CCT CCA ACC CCC CCA TAT TTG GTG GAT  -  126
  22 -  C   T   A   E   S   L   R   T   N   G   S   E   P   P   T   P   P   Y   L   V   D   -   42

 127 - GCC TGG CTG GTC CCA CTC TTC TTT GCC ATT CTG ATG TTA GTT GGG CTA GTG GGC AAC TCT CTG  -  189
  43 -  A   W   L   V   P   L   F   F   A   I   L   M   L   V   G   L   V   G   N   S   L   -   63
                                                                      TMD1
 190 - GTA ATC TAT GTC ATC ATC AGA CAC AAG CCA ATG AGG ACA GTG ACC AAC TAC TAC ATT GCC AAC  -  252
  64 -  V   I   Y   V   I   I   R   H   K   P   M   R   T   V   T   N   Y   Y   I   A   N   -   84

 253 - CTG GCT ACC ACC GAC ATC ATT TTT CTG GTT TGC TGT GTC CCC TTC ACT GCA ATG CTG TAT CCG  -  315
  85 -  L   A   T   T   D   I   I   F   L   V   C   C   V   P   F   T   A   M   L   Y   P   -  105
                                                                          TMD2
 316 - CTT CCA GGC TGG ATC TTT GGA GAA TTC CTG TGT AAA TTT GTC AAT TAC ATC CAA CAG GTC TCA  -  378
 106 -  L   P   G   W   I   F   G   E   F   L   C   K   F   V   N   Y   I   Q   Q   V   S   -  126

 379 - GTG CAA GCC ACG TGC GTC ACC CTG ACC GCC ATG AGT GTG GAC CGG TGG TAC GTG ACC GTG TTT  -  441
 127 -  V   Q   A   T   C   V   T   L   T   A   M   S   V   D   R   W   Y   V   T   V   F   -  147
                                                  TMD3
 442 - CCA CTT CGA TCC CTG CGA CAG CGC ACG CCA CGA GTC GCT GCA AAT GTT AGC ATC AGC ATT TGG  -  504
 148 -  P   L   R   S   L   R   Q   R   T   P   R   V   A   A   N   V   S   I   S   I   W   -  168

 505 - GTT GGT TCC TTC ATT GTC TCC ATC CCG GTG CTG ATT TAC AAC CAG TTA ATA GAA GGC TAT TGG  -  567
 169 -  V   G   S   F   I   V   S   I   P   V   L   I   Y   N   Q   L   I   E   G   Y   W   -  189
        TMD4
 568 - TTC GGT CCA CAA AAG TTC TGC AGC GAG TCC TTC CCC TCT GTG TCC CAT GAG AGA GCC TTC ATC  -  630
 190 -  F   G   P   Q   K   F   C   S   E   S   F   P   S   V   S   H   E   R   A   F   I   -  210

 631 - CTG TAC AAC TTT TTA GTG GTG TAC CTC CTC CCT TTA TTG ACC ATC TTC CTC TGT TAT AGT GCT  -  693
 211 -  L   Y   N   F   L   V   V   Y   L   L   P   L   L   T   I   F   L   C   Y   S   A   -  231
                                             TMD5
 694 - ATG CTT TAC CAG ATG GGA CAC CCC ACT GTG GAG CCA GCT GAT AAC CAC TAC CAG GTA CAG CAA  -  756
 232 -  M   L   Y   Q   M   G   H   P   T   V   E   P   A   D   N   H   Y   Q   V   Q   Q   -  252

 757 - CTG GCA GAA CTC TCT GAA GCT ATG CGA GCC AAG ATC TCT CGT ATG GTG GCC GTC ATA ATT GCT  -  819
 253 -  L   A   E   L   S   E   A   M   R   A   K   I   S   R   M   V   A   V   I   I   A   -  273

 820 - CTG TTC ACT ATC TGC TGG GGT CCA GTG CAG CTT CTG ATC CTC TTC CAA GCC TTT GAC TCC AGC  -  882
 274 -  L   F   T   I   C   W   G   P   V   Q   L   L   I   L   F   Q   A   F   D   S   S   -  294
                    TMD6
 883 - TTT CGG CAC AAT TAC TAT ACC TAT AAG GTG AAG ATT TGG GCT CAC TGC ATG TCC TAT GCC AAT  -  945
 295 -  F   R   H   N   Y   Y   T   Y   K   V   K   I   W   A   H   C   M   S   Y   A   N   -  315

 946 - TCC TCC CTC AAC CCC ATT GTC TAC GCC TTC ATG GGC ACT AAT TTT CGG AAG GCC TTC AAG AAA  - 1008
 316 -  S   S   L   N   P   I   V   Y   A   F   M   G   T   N   F   R   K   A   F   K   K   -  336
        TMD7
1009 - GTT TTC CCA CTG GCC TTT AAG CAG AGG GTG GGT AGC TCC AAC ATT AGT GGC CAG CCC AAT GTC  - 1071
 337 -  V   F   P   L   A   F   K   Q   R   V   G   S   S   N   I   S   G   Q   P   N   V   -  357

1072 - AAC ACC GAG ATG CAG TTT GTC TTA TCA GGA ATG TAG                                      - 1107
 358 -  N   T   E   M   Q   F   V   L   S   G   M   *                                       -  378
```

Fig. S3B

>Indian python KissR4

```
   1 - ATG TCT CCG GAC ACT AGC CCT CGG CTG CCC CTG CGC AGG GCT GCA GAG CCT CCG CCT CCC GAA  -   63
   1 -  M   S   P   D   T   S   P   R   L   P   L   R   R   A   A   E   P   P   P   P   E   -   21

  64 - GCC TCC TGG GCC AGC CAG CTG GAC GAG CCC ACC GTA GCC TGG CAG GGT GAC CCC ATC AGC GCC  -  126
  22 -  A   S   W   A   S   Q   L   D   E   P   T   V   A   W   Q   G   D   P   I   S   A   -   42

 127 - AGG CCC AGC ACA GGC ACC TTC CAG CTT GGG ATG CAG TTC TGG ATC TTT AAC CAC AGC GGA GAA  -  189
  43 -  R   P   S   T   G   T   F   Q   L   G   M   Q   F   W   I   F   N   H   S   G   E   -   63

 190 - GAG ACC TCG CCC CCG TTT CTG ACC GAT GCT TGG CTC GTG CCG GTC TTT TAC GCC CTC ATC ATG  -  252
  64 -  E   T   S   P   P   F   L   T   D   A   W   L   V   P   V   F   Y   A   L   I   M   -   84

 253 - CTG CTG GGC CTG GTG GGG AAC GCC CTG GTC ATC TAT GTC ATC AGC AAG CAC CGC CAG ATG CGC  -  315
  85 -  L   L   G   L   V   G   N   A   L   V   I   Y   V   I   S   K   H   R   Q   M   R   -  105
         TMD1
 316 - ACA GCT ACA AAC TTC TAC ATT GCA AAT TTG GCT ACC ACT GAC ATC ATA TTC TTA GTG TGC TGT  -  378
 106 -  T   A   T   N   F   Y   I   A   N   L   A   T   T   D   I   I   F   L   V   C   C   -  126
                                                                 TMD2
 379 - GTG CCC TTC ACT GCC ACT CTC TAT CCC CTG CCC AGC TGG GTG TTT GGC GAC TTT ATG TGC AAA  -  441
 127 -  V   P   F   T   A   T   L   Y   P   L   P   S   W   V   F   G   D   F   M   C   K   -  147

 442 - TTT GTC AAC TAC TTA CAG CAG GTG ACC GTC CAG GCC ACC TGC ATT ACT CTG ATG GCT ATG AGT  -  504
 148 -  F   V   N   Y   L   Q   Q   V   T   V   Q   A   T   C   I   T   L   M   A   M   S   -  168
                                                                     TMD3
 505 - GTG GAC CGC TGC TAT GCT ACA CTG TAC CCA CTG CAA TCG TTG CGT TAT CGA ACC CCC CAG GTA  -  567
 169 -  V   D   R   C   Y   A   T   L   Y   P   L   Q   S   L   R   Y   R   T   P   Q   V   -  189

 568 - GCC ATG GCT GTC AGC TTT GCT ATT TGG ATT GGT TCC TTC ATT CTC TCA CTA CCC ATG GCT ATG  -  630
 190 -  A   M   A   V   S   F   A   I   W   I   G   S   F   I   L   S   L   P   M   A   M   -  210
                                             TMD4
 631 - TAT CAT CGC ACC GAA AAT GGC TAC TGG TAT GGT TTA CGT ACC TAC TGC ATT GAA GCT TTC ACA  -  693
 211 -  Y   H   R   T   E   N   G   Y   W   Y   G   L   R   T   Y   C   I   E   A   F   T   -  231

 694 - AGC AAG AGC CAG GAG CGC AGC TTT ATC CTC TAC ACT TTC CTG GGC GTT TAT CTA TTG CCT CTG  -  756
 232 -  S   K   S   Q   E   R   S   F   I   L   Y   T   F   L   G   V   Y   L   L   P   L   -  252
                                                                                 TMD5
 757 - CTC ACT ATT TGC TTC TGC TAC TCC ATC ATG CTC AAG CGC ATT GGA CGT CCT GTG GTT GAA CCA  -  819
 253 -  L   T   I   C   F   C   Y   S   I   M   L   K   R   I   G   R   P   V   V   E   P   -  273

 820 - GTG GAC CAT GAT TAC CAG CAA GTG CAA CAT CTC TCT GAA CGC TCC GCT GCC ATG CGA GCG AAG  -  882
 274 -  V   D   H   D   Y   Q   Q   V   Q   H   L   S   E   R   S   A   A   M   R   A   K   -  294

 883 - ATT TCC AAG ATG GTT GTA GTG ATT GTA CTT CTT TTT GCC ATT TGC TGG GGT CCT ATC CAG TTC  -  945
 295 -  I   S   K   M   V   V   V   I   V   L   L   F   A   I   C   W   G   P   I   Q   F   -  315
                                                             TMD6
 946 - TAC CTG CTT TTT CAG GGC TTC TAT CTC CAT TTC CAG GCG AAC TAT GAG ACC TAT AAG ATT AAG  - 1008
 316 -  Y   L   L   F   Q   G   F   Y   L   H   F   Q   A   N   Y   E   T   Y   K   I   K   -  336

1009 - ACA TGG GCT AAT TGC ATG TCT TAT GCT AAC TCT TCC CTC AAC CCC ATT GTT TAT GCT TTC ATG  - 1071
 337 -  T   W   A   N   C   M   S   Y   A   N   S   S   L   N   P   I   V   Y   A   F   M   -  357
                                                 TMD7
1072 - GGA GAC AGT TTC CGC AAA TCC TTC AAG AAG GCG TTC CCC TTC TTC TTC CGC CAG CGT GTA CGG  - 1134
 358 -  G   D   S   F   R   K   S   F   K   K   A   F   P   F   F   F   R   Q   R   V   R   -  378

1135 - GAC AAT GGC ATG CAT TCA GGC TCC CGT AAT GCG GAG ATG AAA TTT GTT ACT GAA GAG ACC TAA  - 1197
 379 -  D   N   G   M   H   S   G   S   R   N   A   E   M   K   F   V   T   E   E   T   *   -  399
```

Fig. S3C

>Painted Turtle KissR1

1 - ATG CGG GGA GCA TCC GCC GGC GCC CAG CTC AAC GCC TCG CTC CTG CTC CCG AAC CGC TCC TCC - 63
1 - M R G A S A G A Q L N A S L L L P N R S S - 21

64 - AAC AGC TCG GAG CCG CGG AGC CCC CCG CGC CTG GTG GAC GCC TGG CTG GTG CCC CTC TTC TTC - 126
22 - N S S E P R S P P R L V D A W L V P L F F - 42

127 - GCC ATG CTG ATG GTG GTG GGG CTG GCG GGC AAC TCC CTG GTC ATC TAC GTG ATC ACC AAG CAC - 189
43 - A M L M V V G L A G N S L V I Y V I T K H - 63

TMD1

190 - AAG CAG ATG AGG ACG GTC ACC AAC TTC TAC ATC GCC AAC CTT GCC ACC ACC GAT ATC ATC TTC - 252
64 - K Q M R T V T N F Y I A N L A T T D I I F - 84

TMD2

253 - CTG GTG TGC TGC GTG CCG TTC ACT GCC ATG CTG TAT CCC CTT CCC GGA TGG ATC TTC GGG GAG - 315
85 - L V C C V P F T A M L Y P L P G W I F G E - 105

316 - TTC ATG TGC AAG TTT GTC AAT TAC ATC CAA CAG GTC TCC GTG CAG GCC ACC TGC GTC ACC CTG - 378
106 - F M C K F V N Y I Q Q V S V Q A T C V T L - 126

TMD3

379 - ACG GCC ATG AGC GTG GAC CGC TGG TAC GTG ACT GTG TTC CCA CTG CGC TCC CTG CGC CAG CGC - 441
127 - T A M S V D R W Y V T V F P L R S L R Q R - 147

442 - ACC CCC CGC ATT GCC GTT GCC ATC AGC CTG GGC ATC TGG ATC TGT TCC TTT ATC GTC TCC GCC - 504
148 - T P R I A V A I S L G I W I C S F I V S A - 168

TMD4

505 - CCG GTG CTG ATG TAC AAC AGG TTG ACA GAG GGC TAC TGG TTC GGG CCG CAG ACC TAT TGC AGT - 567
169 - P V L M Y N R L T E G Y W F G P Q T Y C S - 189

568 - GAG TCC TTC CCC TCC GCC TCC CAC GAG AAG GCC TTT ATC CTT TAC AAC TTC CTG GCA GTC TAC - 630
190 - E S F P S A S H E K A F I L Y N F L A V Y - 210

631 - CTG CTG CCC CTA CTG ACC ATC TGC ATC TGC TAC GTG GCC ATG CTC TAC CAG ATG GGA CAT CCC - 693
211 - L L P L L T I C I C Y V A M L Y Q M G H P - 231

TMD5

694 - ACC GTG GAG CCC ATC GAT AAC AAC TAC CAG GTG CAA CAG CTG GCC GAG CGC TCC GAG GCG ATG - 756
232 - T V E P I D N N Y Q V Q Q L A E R S E A M - 252

757 - CGG GCC AAG ATC TCC CGC ATG GTG GTC ATC ATC GTG GTA CTC TTC ACC GTC TGT TGG GGG CCC - 819
253 - R A K I S R M V V I I V V L F T V C W G P - 273

TMD6

820 - GTG CAG TTC CTC ATT CTC GTC CAG GCC TTC AGC CCC AGC TTC CAG CGC AAC TAC TAC ATC TAC - 882
274 - V Q F L I L V Q A F S P S F Q R N Y Y I Y - 294

883 - AAG GTG AAG ATC TGG GCC CAC TGC ATG TCC TAC ACC AAC TCC TCC ATC AAC CCC ATC GTC TAC - 945
295 - K V K I W A H C M S Y T N S S I N P I V Y - 315

TMD7

946 - GCC TTC ATG GGT GTC AAC TTC AGG AAA GCC TTC AAG AAG GTC TTC CCC TTC ATC TTC AAG CAA - 1008
316 - A F M G V N F R K A F K K V F P F I F K Q - 336

1009 - AAG GTG GGC TGC ACC AAT GCG GGC AAC GCC CAT GTC AAC ACC GAG ATG CAC TTC GTG TCC TCG - 1071
337 - K V G C T N A G N A H V N T E M H F V S S - 357

1072 - GGC ACA TAG - 1080
358 - G T * - 359

Fig. S3D

>Painted Turtle KissR4

```
   1 - ATG GCA GGG TTG CCA AGG ATG GAT GCA GAG CCC TCC CCG TTG GGC GGC TGG CTG GAC AAT GGC -   63
   1 -  M   A   G   L   P   R   M   D   A   E   P   S   P   L   G   G   W   L   D   N   G  -   21

  64 - TCT TGG ACA CCA GTG CCC ACT GCT GCC CCC CAC ATG GAC ATG AGC ACG CCG GCG GGC CCT GGC -  126
  22 -  S   W   T   P   V   P   T   A   A   P   H   M   D   M   S   T   P   A   G   P   G  -   42

 127 - AGG AGG GGG CTG TGG ATC TTT AAT AGC AGT GGG GAG GAC ACG TCA CCC CCA TTC CTG ACG GAT -  189
  43 -  R   R   G   L   W   I   F   N   S   S   G   E   D   T   S   P   P   F   L   T   D  -   63

 190 - GCT TGG CTG GTG CCT CTT TTC TAT GCC CTC GTC ATG CTG CTG GGG CTG GTG GGC AAC TCA CTC -  252
  64 -  A   W   L   V   P   L   F   Y   A   L   V   M   L   L   G   L   V   G   N   S   L  -   84
                                                                      TMD1
 253 - GTC ATC TAC GTG GTG TCC AAG CAC CGG CAG ATG CGC ACA GCC ACC AAC TTC TAC ATC GCA AAC -  315
  85 -  V   I   Y   V   V   S   K   H   R   Q   M   R   T   A   T   N   F   Y   I   A   N  -  105

 316 - CTG GCA ACC ACA GAC ATC ATC TTC CTG GTG TGC TGT GTC CCC TTC ACC GCC ACG CTC TAC CCC -  378
 106 -  L   A   T   T   D   I   I   F   L   V   C   C   V   P   F   T   A   T   L   Y   P  -  126
                             TMD2
 379 - CTG CCC AGC TGG ATC TTC GGT GAC TTC ATG TGC AAA CTC GTC AAC TAC CTG CAA CAG GTG ACA -  441
 127 -  L   P   S   W   I   F   G   D   F   M   C   K   L   V   N   Y   L   Q   Q   V   T  -  147

 442 - GCG CAG GCC ACC TGC ATC ACC CTG ACG GCG ATG AGC ATG GAC CGC TGC TAC GCC ACC CTC TAC -  504
 148 -  A   Q   A   T   C   I   T   L   T   A   M   S   M   D   R   C   Y   A   T   L   Y  -  168
          TMD3
 505 - CCG CTG CAG TCG CTG CGC TAC CGC ACC CCA CGC GTG GCC ATG GGC GTC AGC GTG GCC ATC TGG -  567
 169 -  P   L   Q   S   L   R   Y   R   T   P   R   V   A   M   G   V   S   V   A   I   W  -  189

 568 - ATG AGC TCC TTC CTT CTC TCG CTG CCC ATA GCC ATG TAC CAC CGC ACT GAG GTG GGC TAC TGG -  630
 190 -  M   S   S   F   L   L   S   L   P   I   A   M   Y   H   R   T   E   V   G   Y   W  -  210
        TMD4
 631 - TAT GGG CTG CGC ACC TAC TGC ATC GAG GCC TTC GCC AGC AAG ATC CAG GAG CGC AGC ATC ATC -  693
 211 -  Y   G   L   R   T   Y   C   I   E   A   F   A   S   K   I   Q   E   R   S   I   I  -  231

 694 - CTC TAC ACC TTC CTG GCT GTC TAC CTG CTG CCT CTG CTC ACC ATC TGC CTC TGC TAC TCT GTC -  756
 232 -  L   Y   T   F   L   A   V   Y   L   L   P   L   L   T   I   C   L   C   Y   S   V  -  252
                                                  TMD5
 757 - ATG CTC AAG CGT GTG GGG CGC CCC ATC GTG GAG CCC ATT GAC CAC AAC TAC CAG GTA CAG CAC -  819
 253 -  M   L   K   R   V   G   R   P   I   V   E   P   I   D   H   N   Y   Q   V   Q   H  -  273

 820 - CTG TCC GAG CAC TCT GTC GCC ATG AGG GCC AAG GTG TCC AAG ATG GTG GTG GTG ATC GTG GTA -  882
 274 -  L   S   E   H   S   V   A   M   R   A   K   V   S   K   M   V   V   V   I   V   V  -  294

 883 - CTC TTC ACT GTG TGC TGG GGC CCC ATC CAG CTC TAC CTC CTC TTC CAG GGC TTC TAT GGC AGC -  945
 295 -  L   F   T   V   C   W   G   P   I   Q   L   Y   L   L   F   Q   G   F   Y   G   S  -  315
              TMD6
 946 - TTC CAG GCC AAC TAC GAG ACC TAC AAG ATC AAG ACG TGG GCC AAC TGC ATG TCC TAC GCC AAC - 1008
 316 -  F   Q   A   N   Y   E   T   Y   K   I   K   T   W   A   N   C   M   S   Y   A   N  -  336

1009 - TCC TCC ATC AAC CCC ATC GTC TAC GCC TTC ATG GGC GAC AGC TTC AGG AAG TCC TTC AAG AAG - 1071
 337 -  S   S   I   N   P   I   V   Y   A   F   M   G   D   S   F   R   K   S   F   K   K  -  357
      TMD7
1072 - GCT TTC CCC TTC CTC TTC CGG CGC CGG GTG CGG GAC GGC GCC GTC CTC TCT GGC TCC CGC AAT - 1134
 358 -  A   F   P   F   L   F   R   R   R   V   R   D   G   A   V   L   S   G   S   R   N  -  378

1135 - GCC GAG ATG AAG TTC GTC ACT GAG GAG ACC TAG                                         - 1167
 379 -  A   E   M   K   F   V   T   E   E   T   *                                          -  388
```

Fig. S3E

>Chinese Turtle partial KissR1

```
  1 - AAC CTG GCC AGC ACA GAC ATC ATC TTC CTG GTG TGC TGC GTG CCG TTC ACC GCC GTG CTG TAC  -  63
  1 -  N   L   A   S   T   D   I   I   F   L   V   C   C   V   P   F   T   A   V   L   Y   -  21
                                                                              TMD2
 64 - CCC CTG CCC GGC TGG ATC TTT GGC GAG TTC ATG TGC AAG TTT GTC AAT TAC ATC CAG CAG GTC  - 126
 22 -  P   L   P   G   W   I   F   G   E   F   M   C   K   F   V   N   Y   I   Q   Q   V   -  42

127 - TCC GTG CAG GCC ACC TGC GCC ACC CTG ACG GCC ATG AGC ATG GAC CGC TGG TAC GTG ACC GTG  - 189
 43 -  S   V   Q   A   T   C   A   T   L   T   A   M   S   M   D   R   W   Y   V   T   V   -  63
              TMD3
190 - TTC CCG CTG CGC TCC CTG CGC CTG CGC ACC CCC CGC CTC GCC GCC GCC GTC AGC CTG GGC ATC  - 252
 64 -  F   P   L   R   S   L   R   L   R   T   P   R   L   A   A   A   V   S   L   G   I   -  84

253 - TGG ATC TGT TCC TTT GTC CTC TCC ACC CCG GTG CTG GTG TAC AAC AGG TTG ACA GAG GGC TAC  - 315
 85 -  W   I   C   S   F   V   L   S   T   P   V   L   V   Y   N   R   L   T   E   G   Y   - 105
          TMD4
316 - TGG TTT GGG CCG CAG ACC TAC TGC AGC GAG TCC TTC CCC TCT GCT GCC CAC GAG AAG GCC TTC  - 378
106 -  W   F   G   P   Q   T   Y   C   S   E   S   F   P   S   A   A   H   E   K   A   F   - 126

379 - ACC CTC TAC AAC TTC CTG GCC GTC TAC CTG GTG CCC CTC CTG ACC ATC TGT GTC TGC TAC GTG  - 441
127 -  T   L   Y   N   F   L   A   V   Y   L   V   P   L   L   T   I   C   V   C   Y   V   - 147
                                            TMD5
442 - GCC ATG CTC TAC CAG ATG GGC CAT CCC ACC GTG GAG CCG GTC GAT AAC AAC TAC CAG CAG CTG  - 504
148 -  A   M   L   Y   Q   M   G   H   P   T   V   E   P   V   D   N   N   Y   Q   Q   L   - 168

505 - GCG GAA CGC TCC GAG GCG ATG CGG GCC AAG ATC TCC CGC ATG GTG GCC ATC ATC GTG GTG CTC  - 567
169 -  A   E   R   S   E   A   M   R   A   K   I   S   R   M   V   A   I   I   V   V   L   - 189

568 - TTC ACC ATC TGC TGG GGG CCC GTT CAA TTC CTC CTC CTC CTC CAG GCC TTT GCC CCC CGC TTC  - 630
190 -  F   T   I   C   W   G   P   V   Q   F   L   L   L   L   Q   A   F   A   P   R   F   - 210
                      TMD6
631 - CAG CGC AAC TAC TAC GTC TAC AAG GTG AAG ATC TGG GCC CAC TGC ATG TCC TAC GCC AAC TCC  - 693
211 -  Q   R   N   Y   Y   V   Y   K   V   K   I   W   A   H   C   M   S   Y   A   N   S   - 231

694 - TCC GTC AAC CCC GTC GTC TAC GCC TTC ATG GGG GCC AAC TTC CGG AAA GCC TTC AAG AAG GTC  - 756
232 -  S   V   N   P   V   V   Y   A   F   M   G   A   N   F   R   K   A   F   K   K   V   - 252
      TMD7
757 - TTC CCC TTC ATC TTC AGG CCC AAG GTG GGC TGC GCC AGC GTG GGC CGC GCC CAT GCC AAC ACC  - 819
253 -  F   P   F   I   F   R   P   K   V   G   C   A   S   V   G   R   A   H   A   N   T   - 273

820 - GAG ATG CAC TTC GTC TCC TCC GGC ACC TAG                                              - 849
274 -  E   M   H   F   V   S   S   G   T   *                                               - 282
```

Fig. S3F

>Chinese Turtle KissR4

```
   1 - ATG GCA GAC TCA TTG AGG ATG AAC ACA GAG CCC TCC CTG TCG GAC AGC TGG CTG GAC AAC TGC  -   63
   1 -  M   A   D   S   L   R   M   N   T   E   P   S   L   S   D   S   W   L   D   N   C   -   21

  64 - TCT TGG GCA CCA GTG CCC ACT TCT GCC CTG CAG CTA GAC ATG GGA GCA CTG GGG GAC CCT GGC  -  126
  22 -  S   W   A   P   V   P   T   S   A   L   Q   L   D   M   G   A   L   G   D   P   G   -   42

 127 - AGA AAT GGG CTG TGG ATC TTT AAC AGC AGC GGG GAG GAC ATG TCC CCC CCT TTC CTG ACG GAT  -  189
  43 -  R   N   G   L   W   I   F   N   S   S   G   E   D   M   S   P   P   F   L   T   D   -   63

 190 - GCT TGG CTG GTG CCA CTC TTC TAT GCC CTT GTC ATG CTG CTG GGG CTG GTG GGC AAC TCG CTC  -  252
  64 -  A   W   L   V   P   L   F   Y   A   L   V   M   L   L   G   L   V   G   N   S   L   -   84
                                                          TMD1
 253 - GTC ATC TAC GTG GTG TCC AAG CAC CGG CAG ATG CGC ACG GCC ACC AAC TTC TAC ATC GCA AAC  -  315
  85 -  V   I   Y   V   V   S   K   H   R   Q   M   R   T   A   T   N   F   Y   I   A   N   -  105

 316 - CTA GCG ACC ACG GAC ATC ATC TTC CTG GTG TGC TGT GTG CCC TTC ACC GCC ACG CTC TAC CCC  -  378
 106 -  L   A   T   T   D   I   I   F   L   V   C   C   V   P   F   T   A   T   L   Y   P   -  126
                             TMD2
 379 - CTG CCC AGC TGG GTC TTC GGC GAC TTC ATG TGC AAA CTC GTC AAC TAC CTG CAG CAG GTG ACG  -  441
 127 -  L   P   S   W   V   F   G   D   F   M   C   K   L   V   N   Y   L   Q   Q   V   T   -  147

 442 - GCA CAG GCC ACC TGC ATC ACC CTG ACA GCG ATG AGC ATG GAC CGC TGC TAC GCC ACC CTC TAC  -  504
 148 -  A   Q   A   T   C   I   T   L   T   A   M   S   M   D   R   C   Y   A   T   L   Y   -  168
                 TMD3
 505 - CCG CTG CAG TCG CTG CGC TAC CGC ACC CCC CGC ATC GCC ATG GGC GTC AGT GTG GCC ATC TGG  -  567
 169 -  P   L   Q   S   L   R   Y   R   T   P   R   I   A   M   G   V   S   V   A   I   W   -  189

 568 - GTC GGC TCC TTC CTC CTC TCG CTG CCC ATC GCC ATG TAC CAC CGC ACT GAG GTG GGC TAC TGG  -  630
 190 -  V   G   S   F   L   L   S   L   P   I   A   M   Y   H   R   T   E   V   G   Y   W   -  210
   TMD4
 631 - TAC GGG CTG CGC ACA TAC TGC ATC GAG GCC TTC ACC AGC AAG AGC CAA GAG CGC AGC ATC ATC  -  693
 211 -  Y   G   L   R   T   Y   C   I   E   A   F   T   S   K   S   Q   E   R   S   I   I   -  231

 694 - CTC TAC ACC TTC CTG GTG GTC TAC CTG CTG CCC CTG CTC ACC ATC TGC CTC TGC TAT TCT CTC  -  756
 232 -  L   Y   T   F   L   V   V   Y   L   L   P   L   L   T   I   C   L   C   Y   S   L   -  252
                                             TMD5
 757 - ATG CTC AAG CGG GTG GGG CGC CCC GTC GTG GAG CCC ATT GAC CAC AAC TAC CAG GTG CCA CAC  -  819
 253 -  M   L   K   R   V   G   R   P   V   V   E   P   I   D   H   N   Y   Q   V   P   H   -  273

 820 - CTG TCG GAG CGC TCA GTC GCC ATG AGG GCC AAG GTA TCC AAG ATG GTG GTG GTG ATC GTG GTA  -  882
 274 -  L   S   E   R   S   V   A   M   R   A   K   V   S   K   M   V   V   V   I   V   V   -  294

 883 - CTC TTC ACC GTG TGC TGG GGC CCC ATC CAG CTC TAC CTC CTC TTC CAG GGC TTC TAT GGT GGC  -  945
 295 -  L   F   T   V   C   W   G   P   I   Q   L   Y   L   L   F   Q   G   F   Y   G   G   -  315
             TMD6
 946 - TTC CAG GCT AAC TAT GAG ACC TAC AAG ATC AAG ACG TGG GCC AAC TGC ATG TCC TAT GCC AAC  - 1008
 316 -  F   Q   A   N   Y   E   T   Y   K   I   K   T   W   A   N   C   M   S   Y   A   N   -  336

1009 - TCC TCC ATC AAC CCC ATT GTC TAC GCC TTC ATG GGT GAC AGC TTC AGG AAG TCC TTC AAG AAG  - 1071
 337 -  S   S   I   N   P   I   V   Y   A   F   M   G   D   S   F   R   K   S   F   K   K   -  357
   TMD7
1072 - GCT TTC CCC TTC CTC TTC CGG CGC CGG GTG CGA GAT GGC GCC GTC CTC TCC GGC TCT CGC AAT  - 1134
 358 -  A   F   P   F   L   F   R   R   R   V   R   D   G   A   V   L   S   G   S   R   N   -  378

1135 - GCT GAG ATG AAG TTT GTC ACT GAG GAG ACC TAG                                          - 1167
 379 -  A   E   M   K   F   V   T   E   E   T   *                                           -  399
```

Fig. S3G

>Crocodile KissR1

```
   1 - ATG CGG GCA GTG GGG ATG GAC ACA GTG GGC AGC GCC GGG AAC AAT GCA TCG CTG CTG CCC CCC  -   63
   1 -  M   R   A   V   G   M   D   T   V   G   S   A   G   N   N   A   S   L   L   P   P   -   21

  64 - AAC GGG TCG TGT GGG GCT GGG GGG CTG CTG TGC GGA AAC AGC TCG GGG CCC CCC GGC CCC CCA  -  126
  22 -  N   G   S   C   G   A   G   G   L   L   C   G   N   S   S   G   P   P   G   P   P   -   42

 127 - CGC CTG GTG GAT GCC TGG CTG GTG CCC CTC TGC TTC GCA GCG CTG CTG GTG CTG GGG CTG GCG  -  189
  43 -  R   L   V   D   A   W   L   V   P   L   C   F   A   A   L   L   V   L   G   L   A   -   63
                                                                 TMD1
 190 - GGC AAC TCC CTG GTC ATC TAC GTG ATC TCC AGG CAC CAG CCA ATG AGG ACA GTC ACC AAC TTC  -  252
  64 -  G   N   S   L   V   I   Y   V   I   S   R   H   Q   P   M   R   T   V   T   N   F   -   84

 253 - TAC ATC GCC AAC CTG GCC GCC ACC GAC ATC ATC TTC CTG GTC TGC TGC GTG CCC TTC ACT GCC  -  315
  85 -  Y   I   A   N   L   A   A   T   D   I   I   F   L   V   C   C   V   P   F   T   A   -  105
                                             TMD2
 316 - ATG CTG TAC CCG CTG CCC GGC TGG ATC TTC GGG GAG TTC ATG TGC AAG TTC GTT AAC TAC ATC  -  378
 106 -  M   L   Y   P   L   P   G   W   I   F   G   E   F   M   C   K   F   V   N   Y   I   -  126

 379 - CAG CAG GTC TCC GTG CAG GCC ACC TGC ATG ACA CTG ACG GCC ATG AGC GTG GAC CGC TGG TAC  -  441
 127 -  Q   Q   V   S   V   Q   A   T   C   M   T   L   T   A   M   S   V   D   R   W   Y   -  147
                                 TMD3
 442 - GTG ACC GTG TTC CCA CTG CGC TCC CTG CGC CAG CGC ACC CCC CGC GTC GCC GTC ACC GTC AGC  -  504
 148 -  V   T   V   F   P   L   R   S   L   R   Q   R   T   P   R   V   A   V   T   V   S   -  168

 505 - CTG GGC ATC TGG AGC GGT TCC TTC GTC GTC TCC ATC CCG GTG CTG ACG TAC CAC CAG CTG ACG  -  567
 169 -  L   G   I   W   S   G   S   F   V   V   S   I   P   V   L   T   Y   H   Q   L   T   -  189
                              TMD4
 568 - GAG GGC TAC TGG TTC GGG CCC CAG ACC TAC TGC AGC GAG TCC TTC CCC TCG GTG GCT CAC GAG  -  630
 190 -  E   G   Y   W   F   G   P   Q   T   Y   C   S   E   S   F   P   S   V   A   H   E   -  210

 631 - AAG GCC TTC ATC CTC TAC AAC TTC CTG GCT GTG TAC CTG CTG CCT CTG CTG ACC ATC TGC GTC  -  693
 211 -  K   A   F   I   L   Y   N   F   L   A   V   Y   L   L   P   L   L   T   I   C   V   -  231
                                             TMD5
 694 - TGC TAC ACT GCC ATG CTC CAT CAG ATG GGG CAT CCT GCC GTG GAG CCG GCC GAG AAT GGC TAC  -  756
 232 -  C   Y   T   A   M   L   H   Q   M   G   H   P   A   V   E   P   A   E   N   G   Y   -  252

 757 - CAG GTG CAG CAG CTG GCA GAG CGC TCA GAG GCC TTG CGC GCC CGG ATC TCC CGC ATG GTG GCC  -  819
 253 -  Q   V   Q   Q   L   A   E   R   S   E   A   L   R   A   R   I   S   R   M   V   A   -  273

 820 - ATC ATC GTG GTG CTC TTC ACC ATA TGC TGG GGG CCC ATG CAG TTC CTC ATC CTC CTG CAG GCC  -  882
 274 -  I   I   V   V   L   F   T   I   C   W   G   P   M   Q   F   L   I   L   L   Q   A   -  294
                                       TMD6
 883 - TTC GGG CCC GGC TTC CAG CGC AAC TAC TAC ACC TAC AAG GTG AAG ATC TGG GCC CAC TGC ATG  -  945
 295 -  F   G   P   G   F   Q   R   N   Y   Y   T   Y   K   V   K   I   W   A   H   C   M   -  315

 946 - TCC TAT GCC AAT TCC TCG GTC AAC CCC CTC GTC TAT GCC TTC ATG GGC GCC AAC TTC AGG AAG  - 1008
 316 -  S   Y   A   N   S   S   V   N   P   L   V   Y   A   F   M   G   A   N   F   R   K   -  336
                          TMD7
1009 - GCC TTC AGG AAA GTC TTC CCC TTT GCC TTC AAG CAG CGG GTA GGC AGC ACC GGC GCG GGC AGC  - 1071
 337 -  A   F   R   K   V   F   P   F   A   F   K   Q   R   V   G   S   T   G   A   G   S   -  357

1072 - GCC AAC GTC AAC ACC GAG ATG CAC TTT GTC TCC TCC GGC AAG TAG                          - 1116
 358 -  A   N   V   N   T   E   M   H   F   V   S   S   G   K   *                           -  378
```

Fig. S3H

>Garial KissR1

```
1 - ATG TGG GCA GTG GGG ATG GGC ACA GTG GGC AGC GCC GGG AAC AAT GCA TCG CTG CTG CTT CCC - 63
1 - M W A V G M G T V G S A G N N A S L L L P - 21

64 - CCC AAC GGG TCG TGT GGG GCT GGG GGG CTG CTG TGC GGG AAC AGC TCG GGG CCC CCC AGC CCC - 126
22 - P N G S C G A G G L L C G N S S G P P S P - 42

127 - CCA CGC CTG GTG GAT GCC TGG CTG GTG CCC CTC TGC TTC GCG GCG CTG CTG GTG CTG GGG CTG - 189
43 - P R L V D A W L V P L C F A A L L V L G L - 63
TMD1
190 - GTG GGC AAC TCC CTG GTC ATC TAC GTT ATC TCC AGG CAC CAG CCG ATG AGG ACG GTC ACC AAC - 252
64 - V G N S L V I Y V I S R H Q P M R T V T N - 84

253 - TTC TAC ATC GCC AAC CTG GCC GCC ACC GAC ATC ATC TTC CTG GTC TGC TGC GTG CCC TTC ACT - 315
85 - F Y I A N L A A T D I I F L V C C V P F T - 105
TMD2
316 - GCC ATG CTG TAC CCG CTG CCC AGC TGG ATC TTC GGG GAG TTC ATG TGC AAG TTC ATT AAC TAC - 378
106 - A M L Y P L P S W I F G E F M C K F I N Y - 126

379 - ATC CAG CAG GTC TCC GTG CAG GCC ACC TGC ATG ACG CTG ACG GCC ATG AGC GTG GAC CGC TGG - 441
127 - I Q Q V S V Q A T C M T L T A M S V D R W - 147
TMD3
442 - TAC GTG ACC GTG TTC CCA CTG CGC TCC CTG CGC CAG CGC ACC CCC CGC GTC GCC GTC GCC GTC - 504
148 - Y V T V F P L R S L R Q R T P R V A V A V - 168

505 - AGC CTG GGC ATC TGG AGC GGT TCC TTC GTC GTC TCC GTC CCG GTG CTG ACG TAC CAC CGG CTG - 567
169 - S L G I W S G S F V V S V P V L T Y H R L - 189
TMD4
568 - ACG GAG GGC TAC TGG TTC GGG CCC CAG ACC TAC TGC AGC GAG TCC TTC CCC TCG GTG GCT CAC - 630
190 - T E G Y W F G P Q T Y C S E S F P S V A H - 210

631 - GAG AAG GCC TTC ATC CTC TAC AAC TTC CTG GCT GTG TAC CTG CTG CCT CTG CTG ACC ATC TGC - 693
211 - E K A F I L Y N F L A V Y L L P L L T I C - 231
TMD5
694 - ATC TGC TAC ACC GCC ATG CTC TAT CAG ATG GGG CGT CCT GCC GTG GAG CCG GCT GAG AAT GGC - 756
232 - I C Y T A M L Y Q M G R P A V E P A E N G - 252

757 - TAC CAG GTG CAG CAG CTG GCA GAG CGC TCG GAG GCC TTG CGC GCC CGG ATC TCC CGC ATG GTG - 819
253 - Y Q V Q Q L A E R S E A L R A R I S R M V - 273

820 - GCC ATC ATC GTG GTG CTC TTC ACC ATG TGC TGG GGG CCC ATG CAG TTC CTC ATC CTC CTG CAG - 882
274 - A I I V V L F T M C W G P M Q F L I L L Q - 294
TMD6
883 - GCC TTC GGG CCT GGC TTC CAG CGC AAC TAC TAC ACC TAC AAG GTG AAG ATC TGG GCC CAC TGC - 945
295 - A F G P G F Q R N Y Y T Y K V K I W A H C - 315

946 - ATG TCC TAC GCC AAC TCC TCG GTC AAC CCC CTC GTC TAT GCC TTC ATG GGC GCC AAC TTC AGG - 1008
316 - M S Y A N S S V N P L V Y A F M G A N F R - 336
TMD7
1009 - AAG GCC TTC AGG AAA GTC TTC CCC TTT GCC TTC AAG CAG CGG GTG GGC GGC ACC GGC GCG GGC - 1071
337 - K A F R K V F P F A F K Q R V G G T G A G - 357

1072 - AGC GCC AAC GTC AAC ACC GAG ATG CAC TTT GTC TCC TCC GGC AAG TAG - 1119
358 - S A N V N T E M H F V S S G K * - 378
```
